# Potential biomarkers in early detection of gastric cancer

**DOI:** 10.3389/fphar.2025.1642927

**Published:** 2025-10-31

**Authors:** Supreet Kaur, Ankush Kumar, Rohit Bhatia, Diksha Choudhary, Rajwinder Kaur, Balakumar Chandrasekaran, Wael Abu Dayyih, Majd Nawras Maaita, Wafa Hourani

**Affiliations:** ^1^ Chitkara College of Pharmacy, Chitkara University, Rajpura, Punjab, India; ^2^ Department of Pharmaceutical Sciences, Faculty of Pharmacy, Zarqa University, Zarqa, Jordan; ^3^ Department of Pharmaceutical Chemistry, Faculty of Pharmacy, Mutah University, Al Karak, Jordan; ^4^ Department of Pharmaceutical Sciences, Faculty of Pharmacy, Philadelphia University, Amman, Jordan

**Keywords:** biomarkers, cancer, gastric cancer, signaling pathways, proliferation

## Abstract

The annual burden of gastric cancer (GC) is increasing, highlighting a major threat to global public health. An important contributing factor to the increased fatality of the disease is the late stage at which GC is usually detected. Recent advancements in genomic and molecular studies have spearheaded the discovery of novel biomarkers for early-stage GC. Enabled by metabolomic, genetic, epigenetic, and proteomic signatures, these biomarkers have the potential to change the diagnostic outlook for GC. Such biomarkers would allow the detection of disease in its early stages, thereby improving the quality of life of those affected by this disease and also lowering the mortality rate. This review aims to provide a thorough overview of the novel biomarkers in GCs. Furthermore, this review addresses the mechanism by which these biomarkers are linked to the detection of GC and their possible utilization in clinical settings. This review comprises several novel biomarkers such as heat shock protein family A6 (HSPA6), annexin A11 (ANXA11), cell division cycle 42 (CDC42), fibroblast activation protein-alpha (FAP), hepcidin antimicrobial peptide (HAMP), solute carrier family 25 member 4 (SLC25A4), serpin peptidase inhibitor clade H member 1 (SERPINH1), cystatin B, deleted in malignant brain tumors 1 (DMBT1), nuclear paraspeckle assembly transcript 1 (NEAT1), N6-methyladenosine-related lncRNAs, circular RNAs, and proteinase 3 (PRTN3). Thus, the aim of this review is to gather and incorporate the current state of knowledge on this topic to point out the need for persistent research and innovation in the field of identification of GC biomarkers. This will enable the opportunity for new and more effective strategies for combating GC, which will further reduce its global burden and improve patient survival.

## Graphical Abstract

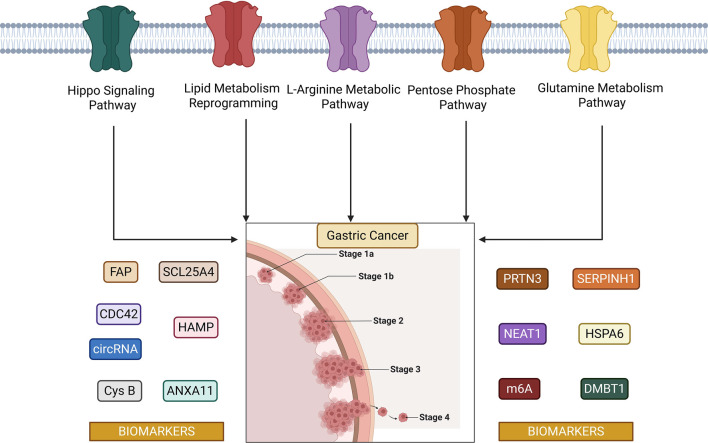

## 1 Introduction

Gastric cancer (GC) is a serious global health concern. With more than a million new cases and 769,000 associated deaths in 2020, GC ranks fourth in the world for mortality among malignancies and fifth for incidence (Mamun et al., 2024). The researchers of the International Agency for Research on Cancer (IARC) forecast that by 2040, the annual load of GC will rise to 1.8 million new cases and approximately 1.3 million deaths (Thrift et al., 2023), depicting a 63% increase in new cases and a 66% increase in deaths. Stomach cancer is the most common cancer in China, Bhutan, Cabo Verde, and Tajikistan, and it affects a large portion of Eastern Asia. Men in eastern Asian nations such as Japan, Mongolia, and the Republic of Korea had the greatest incidence rates, while men in Africa had the lowest incidence rates (Organization, 2025). The incidence and fatality rates differ across various geographic and racial groups. In several South-Central Asian nations, GC is the most often diagnosed malignant tumor and the leading cause of cancer-related death. Additionally, North America and Northern Europe have the lowest incidence rates of GC, while Eastern Asia and Eastern Europe have the highest rates (Sung Y.-K. et al., 2020).

GC is a diverse illness that can present with different phenotypes and molecular profiles (Knight and Allum, 2019). Precancerous lesions, hereditary factors, and *Helicobacter pylori* infection are the main causes of GC (Zhang et al., 2019; Smyth et al., 2020). Men are 2–3 times more likely than women to develop this cancer, and they also die from it at a higher rate (Sung H. et al., 2020). The prevalence of GC varies by geographic region and cultural background, and emerging nations like Iran account for more than 50% of new cases (Bray et al., 2018). In the world, after malignancies of the lung, breast, colorectal, and prostate, GC is the fifth-most prevalent malignant tumor. Although its incidence rate has decreased recently, it is still the third most common cancer-related cause of death globally (Bray et al., 2018; Ferlay et al., 2019). In China, the national population-based cancer registry shows that GC has the third-highest incidence and tumor-associated death rates (Li P. et al., 2024). This conclusion can be linked to the high prevalence of postoperative local recurrence and distant metastasis, the poor treatment outcomes, and the fact that most stomach cancer patients are already at an advanced stage at diagnosis (Siegel et al., 2020).

According to the Ming classification, which corresponds to the Bormann classification (protrusion and ulcer type), the Lauren classification (intestinal and diffuse type), and the WHO classification (papillary adenocarcinoma, adenosquamous carcinoma, squamous cell carcinoma, carcinoid, etc.), GC is separated into infiltrative GC (IGC) and expanding GC (EGC) (Luebke et al., 2005). IGC occurs 61.5% of the time and has a worse prognosis than EGC (Zhang L. et al., 2023). Biomarkers are traits that can be objectively examined and assessed to serve as markers for normal biological processes, pathogenic processes, or pharmacological responses to therapeutic interventions (Smyth et al., 2020). It is understandable why the field of GC biomarkers has drawn so much attention. Some indicators of GC-related DNA, RNA, and exosomes have been found in recent investigations. Despite advancements in diagnostic and therapeutic techniques, the prognosis for GC patients remains poor, largely due to late-stage diagnosis and high rates of recurrence and metastasis. In recent years, the exploration of molecular biomarkers has gained significant attention for their potential to improve early detection, monitor disease progression, and guide personalized treatment strategies (Hanash et al., 2011; Sethi et al., 2013). Biomarkers are measurable indicators of biological or pathological processes and can be detected in tissue, blood, or other bodily fluids (Ahmad et al., 2023). They offer valuable insights into the molecular alterations involved in gastric tumor initiation, progression, immune evasion, and response to therapy (Carlomagno et al., 2017).

Conventional serum markers often fail to detect GC at curable stages or distinguish it from benign conditions, resulting in missed opportunities for timely intervention. Conventional serum tumor markers used in GC, such as CEA, CA19-9, and CA72-4, are constrained by low sensitivity and specificity, particularly for early-stage disease. For instance, positive detection rates for early GC are often below 10%, even when combining markers severely limiting early diagnosis (Yu et al., 2016; Feng et al., 2017). To overcome these limitations, novel biomarkers are being actively explored, including genomic, epigenetic, transcriptomic, proteomic, metabolomic, and liquid biopsy-based candidates. Recent studies have identified promising biomarker classes such as circulating tumor DNA (ctDNA), long non-coding RNAs (lncRNAs), circular RNAs (circRNAs), exosomal miRNAs, DNA methylation markers, and immune checkpoint molecules, many of which offer enhanced diagnostic, prognostic, and predictive value (Jelski and Mroczko, 2022; Jiang et al., 2022). However, challenges remain in their clinical translation. Most novel biomarkers lack validation in large-scale, multicenter studies, and few have progressed to routine clinical use (Hong and Liu, 2022; Romańczyk et al., 2024). In addition, standardization of assay techniques, inter-individual variability, and cost-effectiveness remain significant barriers. It is anticipated that the development of these biomarkers will have a significant impact on the progression of cancer, the choice of effective therapy approaches, and effective follow-up programs (Smyth et al., 2020).

This review aims to provide a comprehensive overview of tissue, salivary, and urine-based biomarkers for GC, highlighting recent advances, clinical relevance, and future directions. By critically evaluating the landscape of GC biomarker research, this review addresses the pressing clinical need for non-invasive, sensitive, and specific biomarkers that can improve early detection, risk stratification, and treatment outcomes in GC.

## 2 Signaling pathways associated with GC

**FIGURE 1:**
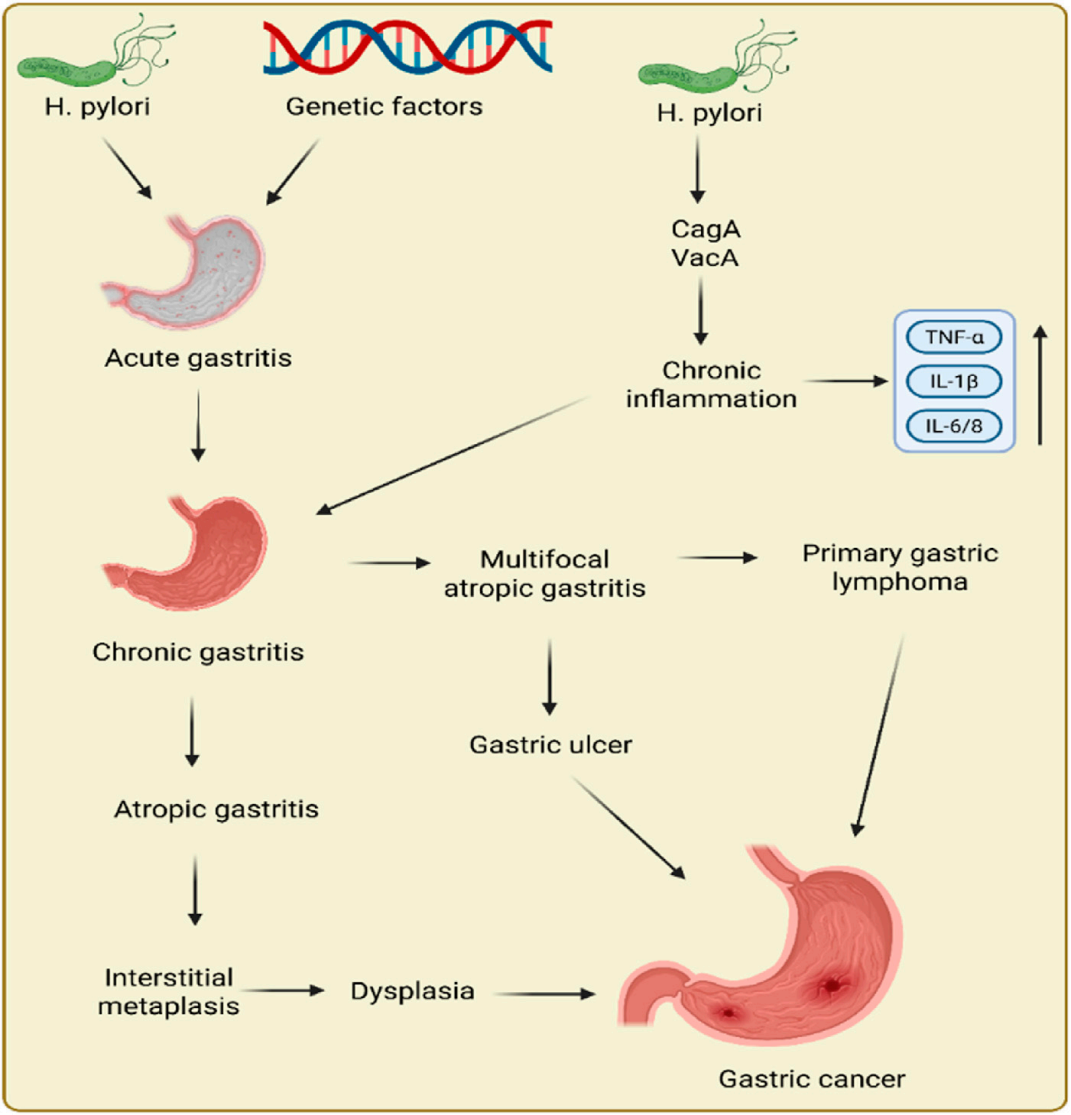
Pathogenesis of gastric cancer.

Epidemiologic and experimental data support the causal role of *H. pylori* infection in GC. Several factors interact to determine whether the infection will cause multifocal atrophic gastritis or non-atrophic gastritis. The latter is the precancerous cascade’s first stage. Robin Warren and Barry Marshall received the Nobel Prize in Physiology or Medicine in 2005 for their revolutionary study connecting infection of *H. pylori* to GC and peptic ulcer (Charitos et al., 2021). It has been established that the potential of *H. pylori* to induce GC is correlated with its virulence, which is mostly influenced by the vacuolating toxin VacA and cytotoxin CagA (Sharndama and Mba, 2022). Intestinal metaplasia, serious gastritis, GC, and cytotoxin CagA have all been associated with vacAs1m1- and CagA-positive bacterial genotypes (Kishk et al., 2021). VaccinAs2m2 genotypes that are CagA-negative cause less infection, they cause a milder form of non-atrophic gastritis, and neoplastic outcomes are not always the consequence (Park J. Y. et al., 2018). In addition to *H. pylori,* there are various other factors, such as radiation, genetic factors, hypoxia, etc., that can lead to the development of GC. Figure 1 displays the pathogenesis of GC.

Several metabolic and signaling pathways are intricately involved in the pathogenesis of GC, and their dysregulation often results in molecular alterations that can be exploited as potential diagnostic and prognostic biomarkers. Rather than describing these pathways in isolation, it is essential to understand how specific changes within them give rise to quantifiable molecular signatures. For instance, lipid metabolism reprogramming is a recognized hallmark of cancer that supports membrane biosynthesis, energy storage, and signaling in rapidly proliferating tumor cells (Yang et al., 2023). In GC, upregulation of enzymes such as fatty acid synthase (FASN) and transcription factors like SREBP1 has been reported, and both are emerging as tissue-based biomarkers associated with tumor progression and poor prognosis (Fernandez et al., 2020). Similarly, the glutamine metabolism pathway is frequently altered in GC to meet the high biosynthetic demands of cancer cells. Increased expression of glutaminase (GLS1) and the glutamine transporter SLC1A5 has been observed in tumor tissues, suggesting their potential utility as metabolic biomarkers for early GC detection (Zhong et al., 2023). The pentose phosphate pathway (PPP) plays a vital role in maintaining redox balance and supplying nucleotides for DNA synthesis. Overexpression of glucose-6-phosphate dehydrogenase (G6PD) in GC is associated with oxidative stress adaptation and tumor aggressiveness, positioning it as a candidate prognostic biomarker (Zhang C. et al., 2014). The L-arginine metabolic pathway, important in modulating immune cell function and nitric oxide production, has also been implicated in GC. Biomarkers such as arginase-1 (ARG1) and argininosuccinate synthase 1 (ASS1) are differentially expressed and contribute to immunosuppressive tumor microenvironments, offering insight into immune evasion mechanisms (Chen et al., 2021). Furthermore, the Hippo signaling pathway, which controls cell proliferation, contact inhibition, and apoptosis, is commonly dysregulated in GC. Aberrant activation of YAP1, the key transcriptional co-activator of this pathway, promotes oncogenic transcriptional programs and is being evaluated as both a tissue and circulating biomarker for prognosis (Zhu et al., 2015; Messina et al., 2023). By establishing these molecular connections between dysregulated pathways and specific biomarkers, this section underscores the relevance of pathway-derived biomarker discovery and strengthens the translational value of such markers in the early detection and clinical management of GC.

The following section provides a visual representation and detailed breakdown of these key pathways, highlighting the molecular alterations involved and their associated biomarkers, thereby emphasizing the mechanistic basis of their relevance in GC detection and progression.

### 2.1 Hippo signaling pathway

**FIGURE 2:**
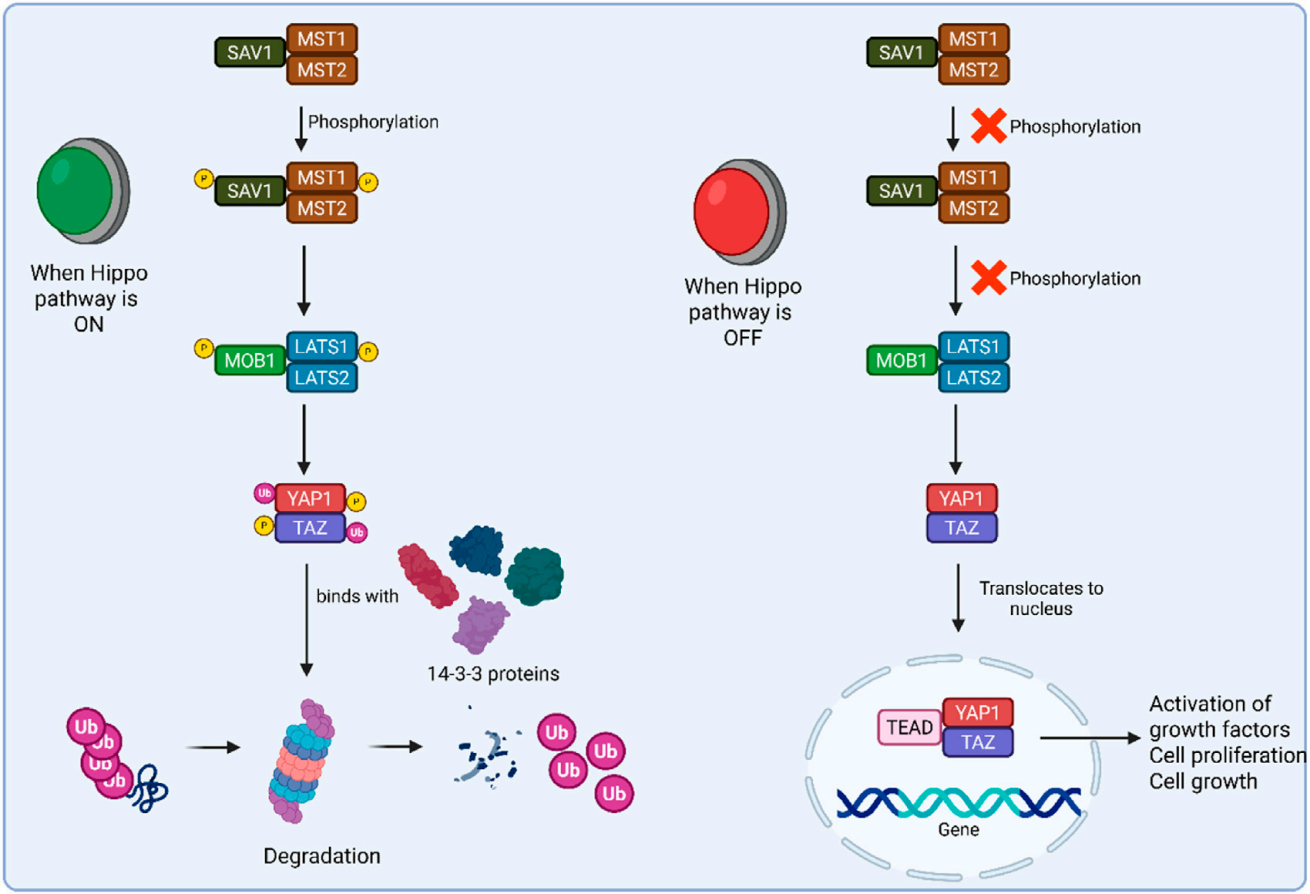
Schematic representation of Hippo on and Hippo off pathways.

A tumor-suppressive mechanism called the Hippo signaling pathway controls tissue growth and restricts the size of organs by preventing proliferation, encouraging apoptosis, and preventing cell growth (Saadh et al., 2025). Once inactivated, the pathway’s downstream elements, like Yes-associated protein 1 (YAP1) and transcriptional co-activator with PDZ binding motif (TAZ), become active and ultimately cause tumorigenesis (Zhao et al., 2023). Under typical conditions, Last1, WW45, Mst1, Mob, and the upstream molecules of the Hippo signaling pathway in mammals frequently create a conserved kinase cassette (Xiao and Dong, 2021). In response to cell density, these molecules can cause phosphorylation and deactivate the YAP/TAZ complex, which is present on a variety of HxRxxS motifs, the most important of which include TAZ S89 and YAP S127. They act as a 14-3-3-binding site and control nucleus-cytoplasmic translocation (Pan, 2010). The transcriptional enhancer factor TEF-1 (TEAD) family of transcription factors will work in conjunction with YAP to control cell development and apoptosis after it enters the nucleus. Figure 2 displays the Hippo on and off pathway in cancer. Three more core molecules are phosphorylated by Mst1 in mammals. Mst1 phosphorylates Lats1 on the hydrophobic motif and activation loop and may involve auto-phosphorylation (Zhao et al., 2008). The shifting of the cytoplasm-nucleus is important in cell growth regulation in response to density and cell-to-cell contact. The nuclear translocation of YAP/TAZ caused by the inactivation of the Hippo signaling system promoted cell proliferation, suppressed cell apoptosis, and ultimately resulted in cancer (Dong et al., 2019).

### 2.2 L-Arginine metabolic pathway

**FIGURE 3:**
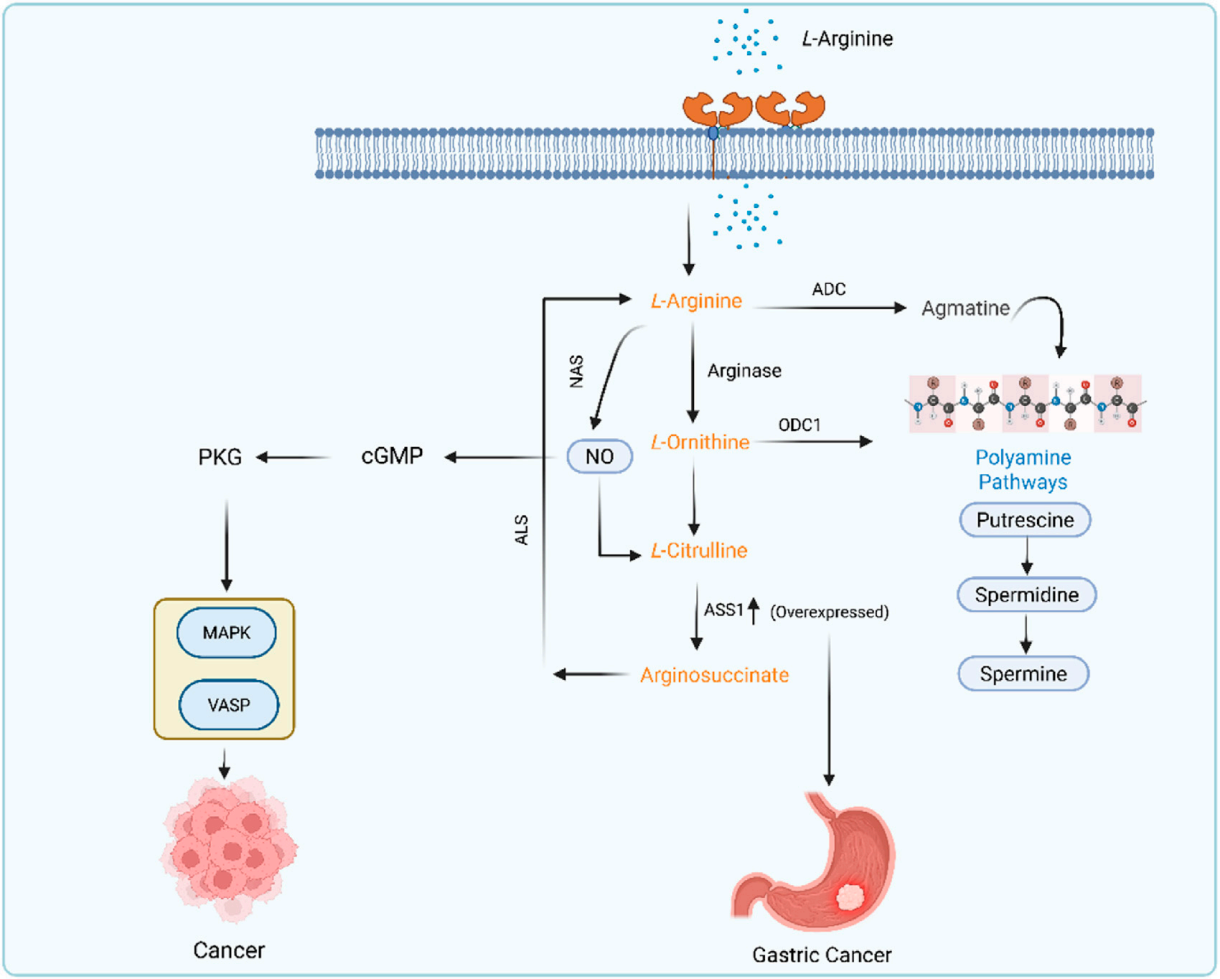
L-arginine metabolic pathway in GC.

Nearly every cell contains amino acids, which are fundamental building blocks of cells. One of the main causes of tumor formation and incidence is metabolic imbalance. The amino acid metabolism patterns give scientists crucial information to comprehend the molecular pathophysiology of malignancies (DeBerardinis and Thompson, 2012). Arginine is a non-essential amino acid that is obtained from dietary products (such as meat and fish) or the intestinal–renal axis (endogenous synthesis) (Wu, 2013). Arginine contributes to the synthesis of protein and acts as a precursor for the various biological molecules such as NO, proline, and urea (THOMPSON, 1980). Arginine and all these substances are continuously involved as sensitive markers in cancer progression, migration, invasion, and angiogenesis (Lind, 2004). Both healthy and malignant cells need the non-essential amino acid arginine for fundamental biological functions like polyamine and protein production (Choi and Coloff, 2019). Thus, arginine has a significant impact on the growth of malignancies. The metabolic pathways of L-arginine are frequently found to be dysregulated in GC, along with many other cancers such as prostate and breast cancer (Feldmeyer et al., 2012). The number of T cells and their functions are impacted by dysregulated metabolism of arginine (Szefel et al., 2019). Insufficient levels of arginine will significantly lessen the impact of T cell-tumor antigen interaction, which will affect the immune system. Furthermore, the lack of arginine increases GC risk due to more severe *H. pylori* infection (Pirzadeh et al., 2022). Arginine is used by the human body as a starting material to produce nitric oxide (NO), ornithine, and agmatine (Wu et al., 2021). Each of these molecules is made through specific enzyme-catalyzed reactions. Arginine is a versatile amino acid that is metabolized into NO and citrulline with the help of nitric acid synthase (NAS) enzyme. Arginine is also metabolized into ornithine and urea by a manganese-containing enzyme called arginase. Arginine is also metabolized into a naturally occurring chemical substance called agmatine with the help of arginase decarboxylase enzyme (ADC) (Grillo and Colombatto, 2004). These two, agmatine and ornithine, act as major resources for the putrescine (Satriano, 2004). Putrescine is a precursor of polyamines such as spermidine and spermine (Qiao et al., 2024). These polyamines are vital for inflammation, cell proliferation, and tumorigenesis. The L-arginine metabolism in cancer is shown in Figure 3.

### 2.3 Pentose phosphate pathway (PPP)

**FIGURE 4:**
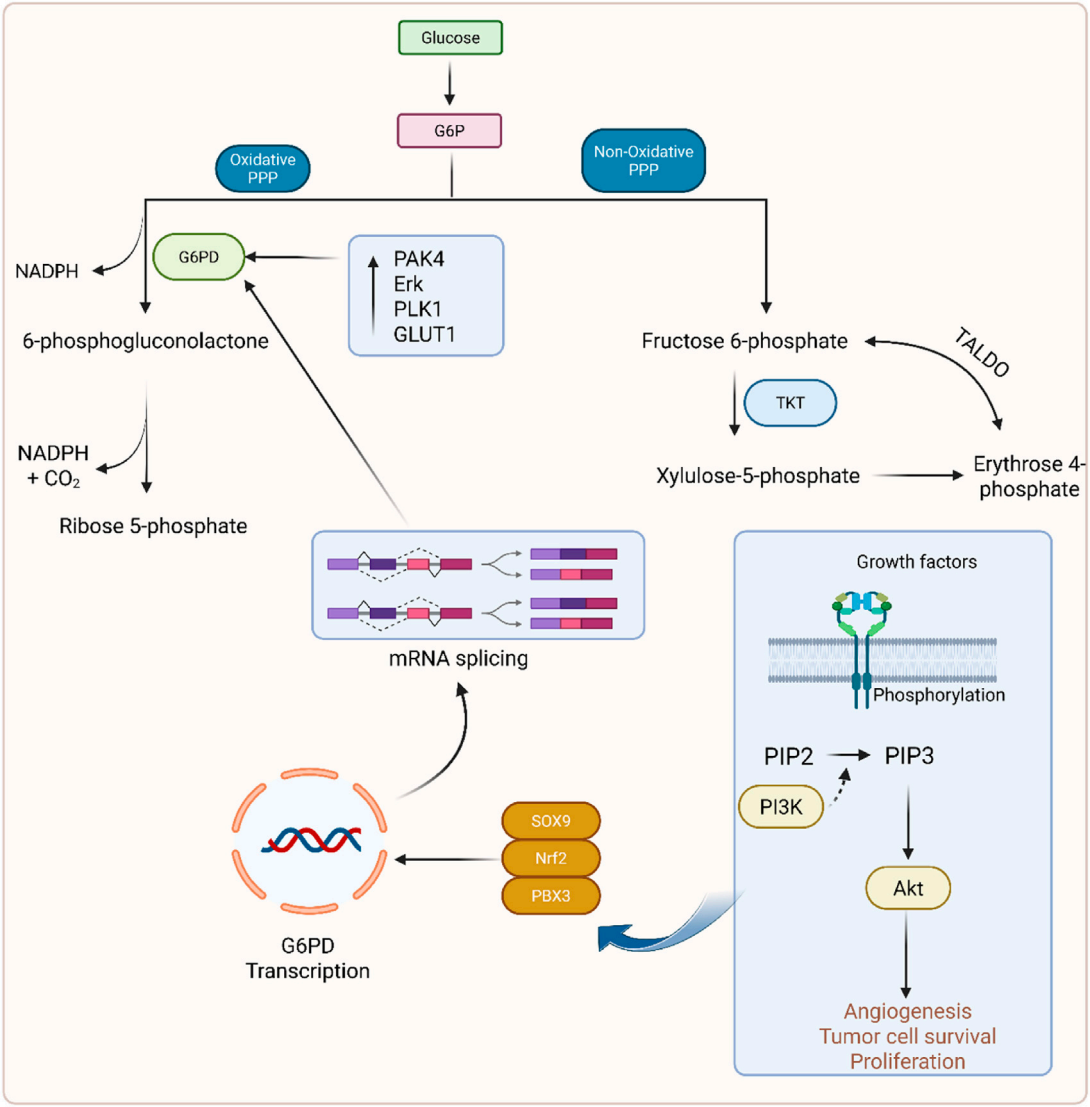
Mechanistic illustration of the phosphate pentose pathway in GC.

PPP is also known as the hexose monophosphate shunt and the phosphogluconate pathway (Sharkey, 2021). This pathway is composed of two sub-pathways, oxidative and non-oxidative PPP (Bradshaw, 2019). In oxidative PPP, two molecules of NADPH are formed along with carbon dioxide in the second step and non-oxidatively form an amino acid (Stincone et al., 2015). Glucose enters the cell through the glucose transporter and, depending on its cellular need, is diverted into respective pathways as glucose-6-phosphate (G6P). The oxidative PPP phase is also referred to as the irreversible phase, which generates NADPH. This G6P is converted into fructose-6-phosphate and 6-phosphoglucolactone in the presence of glucose-6-phosphate dehydrogenase (G6PD). G6PD is found to be overexpressed in cancer cells, which leads to poor survival (Kathagen-Buhmann et al., 2016; Ju et al., 2017). Studies showed that PPP flux modulates cancer directly or indirectly (Patra and Hay, 2014; Weber, 2016). PAK4, Erk, and PLK1 cause direct phosphorylation of G6PD and promote cancer growth (Zhang X. et al., 2017). Pik1 is another kinase that also phosphorylates the G6PD and enhances the PPP flux by promoting the formation of a G6PD dimer to increase the cancer progression (Ma X. et al., 2017). Reduced expression of Rev-erb increased the proliferation of GC (Tao et al., 2019). The PI3k/Akt signaling pathway upregulates SOX9, causing mRNA splicing that results in GC (Xia et al., 2023). PLOD1 increased the GC growth through upregulation of the SOX9/PI3k/Akt/mTOR pathway (Zhang Y. et al., 2021). TKT and TALDO are the two main enzymes that are important for reaction interconversion in non-oxidative PPP (Stincone et al., 2015). It has been found that TALDO is highly overexpressed in gastric adenocarcinoma (Kočevar et al., 2012). PPP is displayed in Figure 4.

### 2.4 Glutamine metabolism pathway

**FIGURE 5:**
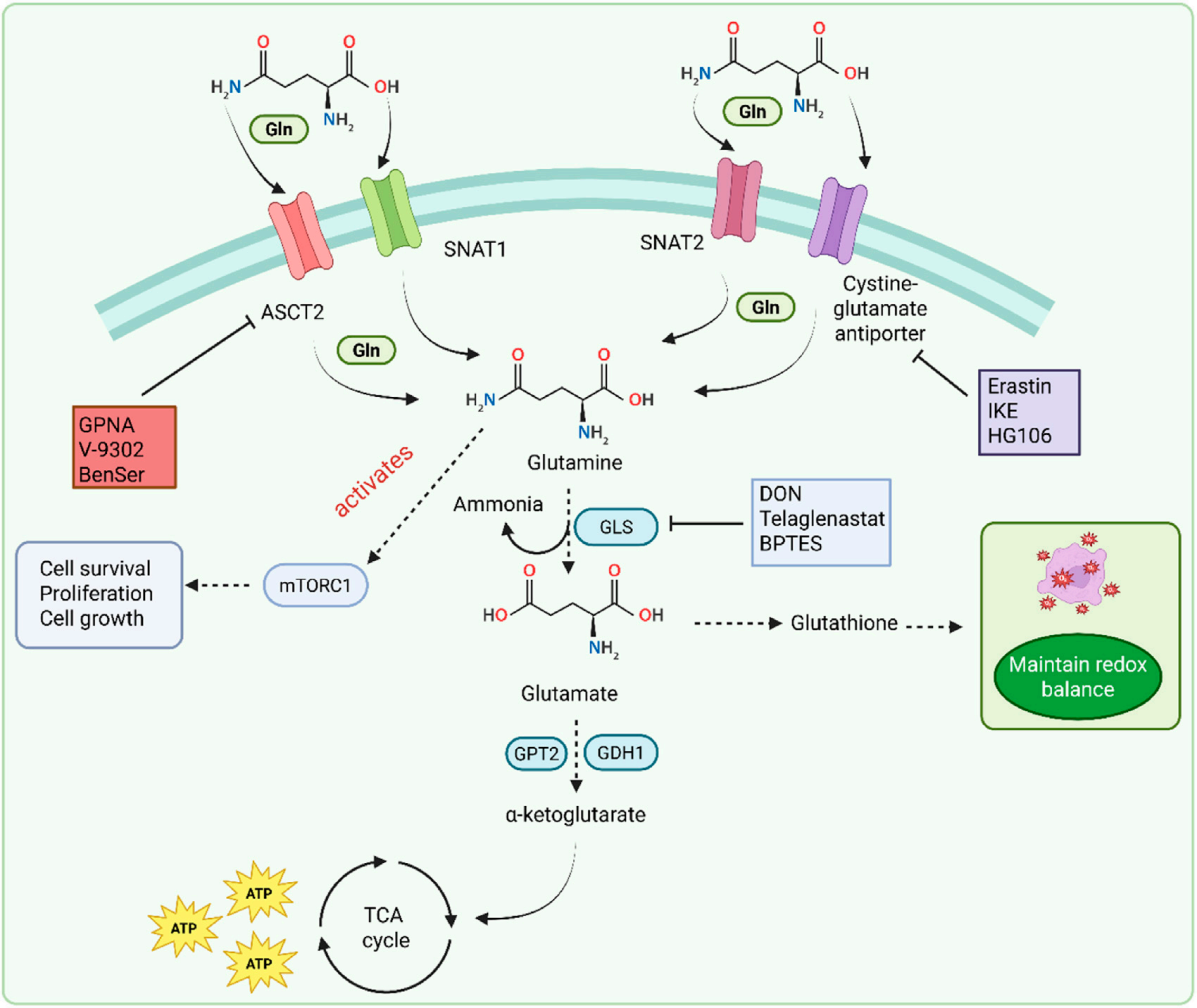
Glutamine metabolism pathway in GC.

The glutamine metabolic pathway plays an important role in cancer progression. Glutamine is an essential amino acid present in cancer cells (Li and Le, 2018). It is present in higher concentration with a range of 0.5–0.6 µM in plasma. It serves as a key nutrient for dividing cancer cells and acts as a source of carbon and nitrogen. Gln is also involved in protein synthesis, nucleotide synthesis, and other processes (Li et al., 2023). Under normal conditions, the body produces enough Gln, but during some physiological or other conditions, such as cancer, its demands increase significantly. Gln enters the cells through various transporters of solute carriers. These transporters include alanine-serine-cysteine transporter 2 (ASCT2), serotonin N-acetyltransferase 1 (SNAT1), serotonin N-acetyltransferase (SNAT2), and cysteine glutamate antiporter (Pochini et al., 2014). Three of these four transporters, ASCT2, SNAT1, and SNAT2, have a main role in cancerous cells. ASCT2 regulates the optimal growth (Marshall et al., 2017), and SNAT1 and SNAT2 help in overall glutamine uptake (Bröer et al., 2016). Gln enters the mitochondria, is converted into glutamate by the glutaminase (GLS) enzyme, and forms ammonia. This converted glutamate is converted into α-ketoglutarate by glutamic-pyruvic transaminase 2 (GPT2) and glutamate dehydrogenase 1 (GDH1). Gln contributes to ATP production by delivering Gln carbon in the TCA cycle. Gln-derived glutamate is converted into glutathione and participates in the maintenance of redox balance (Son et al., 2013). Gln also activates the mTORC1 by simultaneous efflux of the amino acid leucine. This leucine and Gln translocate the mTORC1 to the lysosome, which initiates many processes such as cell survival, proliferation, and cell growth. Skp2 is associated with approximately 50% of patients with GC (Gstaiger et al., 2001; Tanaka et al., 2015). The authors found Skp2 to be a negative regulator of dependent mTORC1. It influenced the Skp2 activity, which helps in phosphorylation mediated by mTORC1 (Fingar, 2015; Jin et al., 2015). A study by Geng et al. found that the Skp2 protein level is regulated by mTORC1 in GC cells (Geng et al., 2017). Phosphorylation of Skp2 by mTORC1 was important for proto-oncogenic functions in GC. Skp2 was found to be expressed in 68 of 83 cases with high mTOR expression (Geng et al., 2017). The signaling pathway is illustrated in Figure 5.

### 2.5 Lipid metabolism reprogramming

**FIGURE 6:**
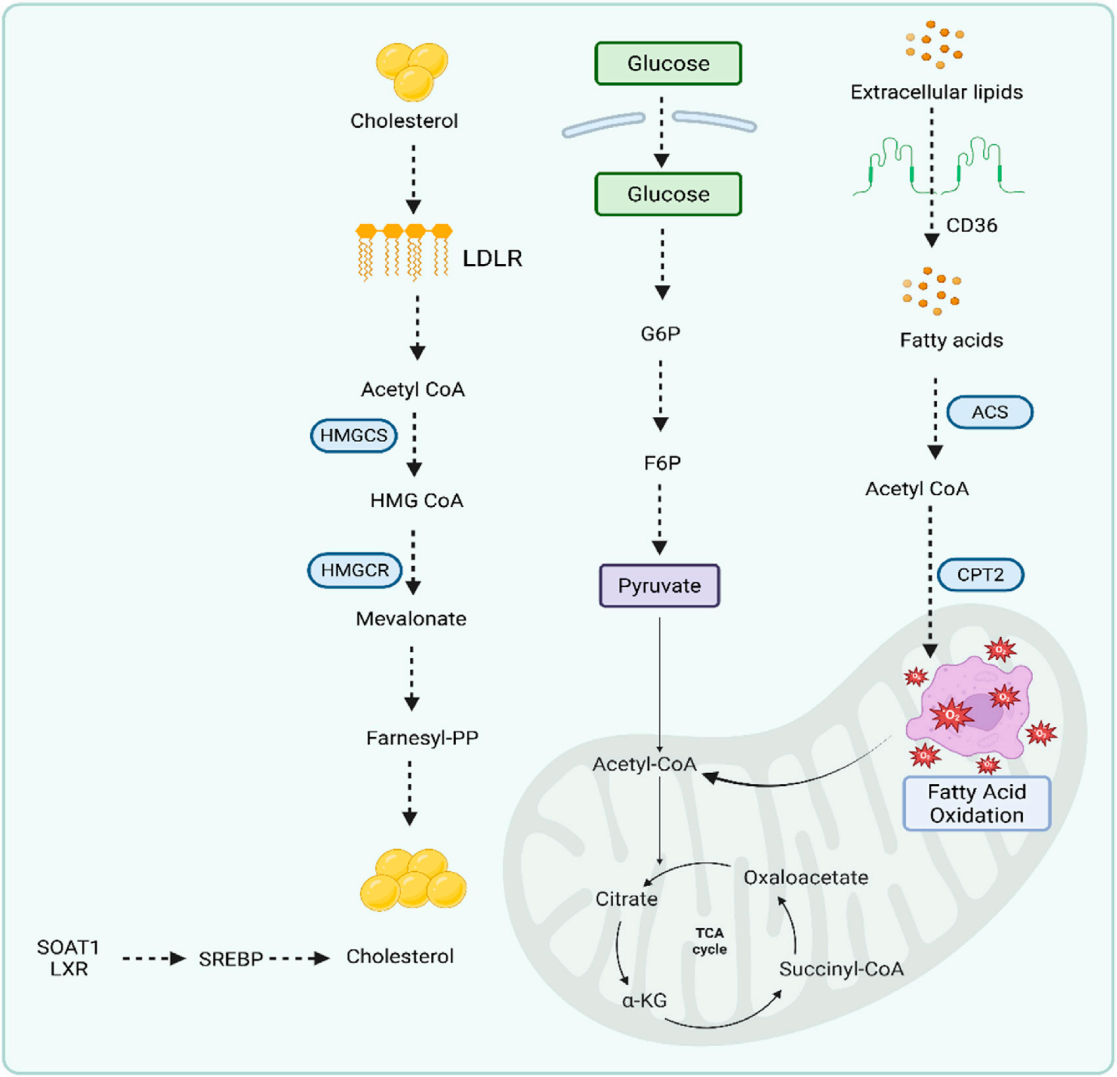
Lipid metabolic programming pathway in gastric cancer.

GC is one of the most common malignant tumors (Bray et al., 2018). It originates in epithelial cells and exhibits a Warburg effect (Liberti and Locasale, 2016). Previous studies showed that under aerobic conditions, tumor tissue metabolizes glucose into lactate in cancer cells compared to normal tissues (Warburg et al., 1927; Liberti and Locasale, 2016). This phenomenon is called the Warburg effect (Warburg et al., 1927). The metabolic alterations are not yet clear. However, changes or alterations in cancer cells could be associated with changes in gene expression. This effect increases the glucose uptake, glycolysis, and the metabolism of pyruvate to lactic acid (Feron, 2009). Lipid metabolic reprogramming depends upon three factors in GC: non-coding RNA, enzymes involved in glycolysis, and mitochondrial proteins. Alterations in these factors create a difference between cancer cells and normal cells. In normal cells, lipids are present in a balanced state for maintenance and membrane synthesis. In the case of gastric cancerous cells, cells take lipids from CD36 receptors and depend upon a *de novo* synthesis pathway. It is also obtained from naturally forming cholesterol through the mevalonate pathway in the synthesis of cholesterol. Although the proper regulatory mechanism in GC is not fully understood, some of the endogenous factors, such as FASN and ACC, are involved in the generation of GC (Nannini et al., 2020). FASN is mainly responsible for proliferation and drug resistance in GC (Ito et al., 2014). LXRs are also involved in the GC through lipid metabolic disorders (Wang et al., 2019). Figure 6 displays the lipid metabolic programming in GC.

## 3 Novel diagnostic biomarkers of GC

**FIGURE 7:**
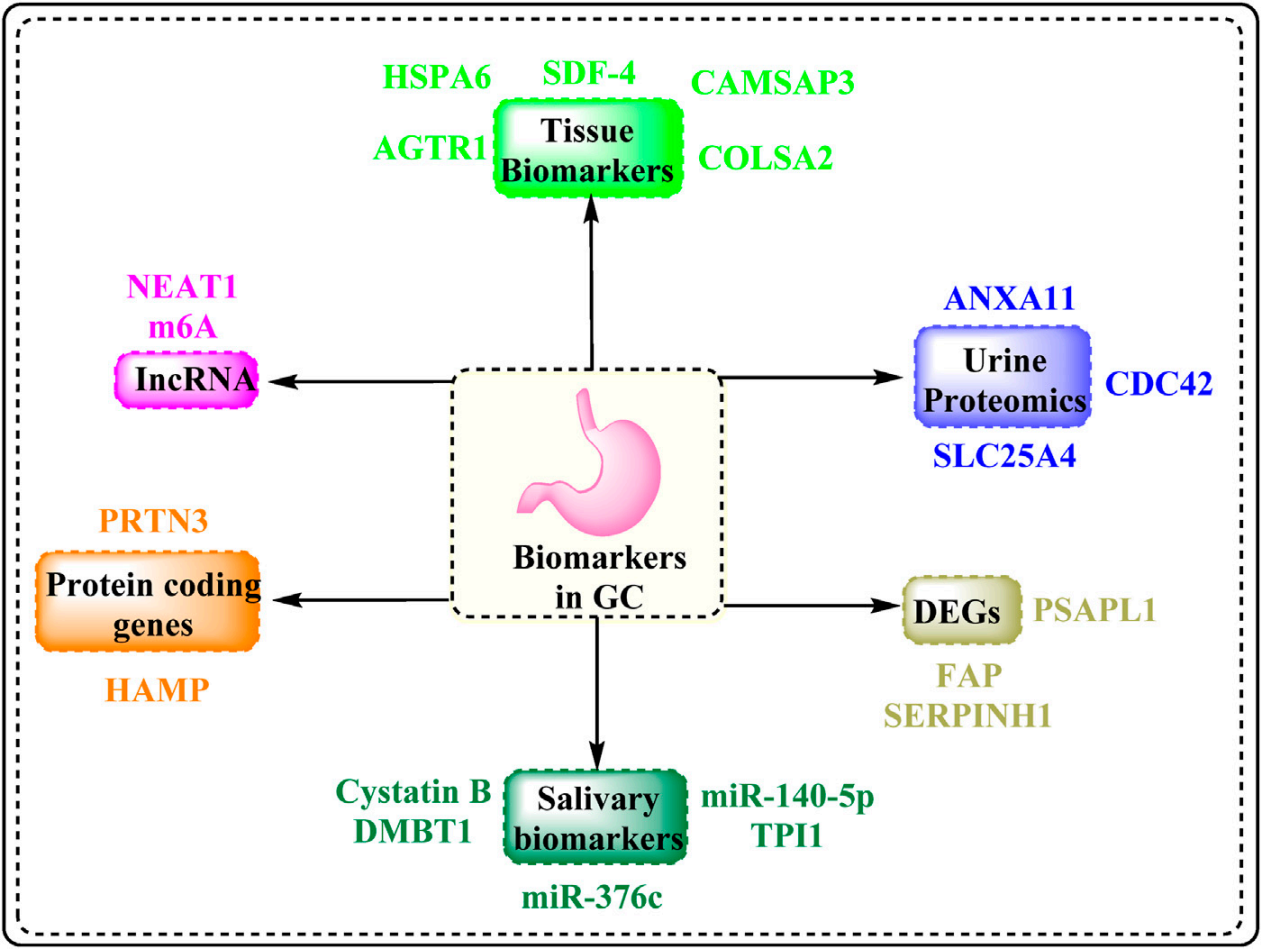
Biomarkers associated with GC.

According to the National Cancer Institute, a biomarker is a biological molecule present in bodily fluids such as blood or tissues that indicates a disease or a normal or dysfunctional process. It is also called a signature molecule or a molecular marker. In this review, novel diagnostic biomarkers are defined as those that have been identified within the past 5–7 years through high-throughput techniques such as transcriptomics, proteomics, and integrative bioinformatics, and that exhibit potential for clinical translation based on recent preclinical or patient-derived data. These biomarkers encompass diverse molecular categories. Differentially expressed genes such as FAP, SERPINH1, and the newly reported PSAPL1 are involved in tumor progression, extracellular matrix remodeling, and immune response modulation. lncRNAs, including NEAT1 and m6A-related lncRNAs, play regulatory roles in gene expression, epithelial–mesenchymal transition, and chemoresistance. circRNAs, which are known for their high stability and tissue specificity, have been implicated in GC by acting as miRNA sponges or regulators of transcription. In addition, protein-based biomarkers such as HSPA6, ANXA11, CDC42, HAMP, SLC25A4, Cystatin B, DMBT1, and PRTN3 have emerged from tissue, salivary, and urinary proteomic studies, showing diagnostic and prognostic potential. Biomarkers were selected based on their recent discovery in the GC research landscape, their significant differential expression between cancerous and normal tissues or fluids, their mechanistic involvement in GC pathogenesis, and supporting evidence of their diagnostic, prognostic, or therapeutic potential from experimental or clinical studies. Figure 7 displays the various categories of GC biomarkers compiled in the present manuscript.

### 3.1 Tissue biomarkers

Tissue biomarkers are molecules that are differentially expressed in tumor tissues compared to normal tissues and can provide valuable diagnostic, prognostic, and therapeutic information (Sarhadi and Armengol, 2022). In GC, tissue-based molecular signatures have been extensively explored due to their direct reflection of the tumor microenvironment and cellular behavior (Del Arco et al., 2024). Advances in transcriptomic and proteomic profiling have enabled the identification of several such biomarkers that show promise for early detection and disease monitoring. In this section, we discuss key tissue biomarkers identified in recent studies, including HSPA6, SDF-4, COL5A2, AGTR1, CAMSAP2, COL1A1, SPARC, and P4HA3, among others. These biomarkers have been reported to be significantly upregulated or altered in GC tissues and are functionally associated with extracellular matrix remodeling, angiogenesis, inflammation, and cellular proliferation. Further evidence from clinical datasets and experimental models supports their potential utility in distinguishing malignant from non-malignant gastric tissue and predicting disease progression. The following subsections elaborate on these biomarkers individually, highlighting their expression patterns, molecular functions, and clinical relevance.

#### 3.1.1 HSPA6

Heat shock proteins (HSPs) were initially identified in *Drosophila* salivary gland cells in 1962 (Sarkar and Roy, 2017). They are a part of a highly conserved and large protein family that is often expressed throughout a range of physiological and stressful situations, including carcinogenesis. The superfamily of HSPs can be broken down into HSP110, HSP90, HSP70, HSP60, HSP40, and a family of tiny HSPs based on molecular weight (Hoter et al., 2018). Located on human chromosome 1q23.3 cytogenetically, 70-kDa heat shock protein 6 (HSPA6) (OMIM: 140555) encodes a protein of 70 kDa. As a stress-induced heat shock gene, HSPA6 was primarily discovered by Leung et al. in 1990 (Shen et al., 2021). In particular, the HSP70 family can shield cells from stress (Beere and Green, 2001). Because they are so effective, they stand for the most conspicuously conserved family (Hill et al., 2013). In humans, 15 members belonging to the HSP70 family have been found in various locations, indicating a variety of site-specific biological roles (Rosenzweig et al., 2019).

Many tumors have high levels of HSP70 expression, which accelerates the development of malignancy by preventing apoptosis, avoiding cellular senescence processes, impairing immunology, and encouraging angiogenesis (Albakova et al., 2020). High levels of HSP70 protein expression are linked with an unfavorable clinical outcome in a variety of malignant tumors (Calderwood et al., 2006). HSPA6 is a potential autosomal-recessive protein that contributes to the spectrum of VATER/VACTERL malformations (Calderwood et al., 2006). Cyto-protection is hypothesized to be a function of HSPA6 (Noonan et al., 2007). There are at least 13 members of the HSP70 family, and HSPA6 (HSP70B′) stands out as being unusual in terms of evolution because it is not conserved in rodents (Hageman et al., 2011). The invasion, migration, and growth of cells in triple-negative breast cancer were decreased by overexpression of HSPA6 (Shen et al., 2021). It was found that HSPA6 is not necessary for withaferin A-mediated suppression of breast cancer migration or apoptosis/autophagy (Hahm et al., 2021). By using RNA sequencing, it was possible to identify HSPA6, the TQ-targeted gene, for functional TNBC (triple-negative breast cancer) inhibition. Because HSPA6 is a “strictly inducible gene,” it cannot be expressed in a healthy or normal state of affairs (Noonan et al., 2007; Shen et al., 2021). CSE strongly but transiently induces HSPA6 (Zhang L. et al., 2023).

According to research, cigarette smoke robustly induces HSPs, particularly HSPA6, in Jurkat cells and DLD-1 at levels that prevent these cells from succumbing to oxidative stress or cytokine-induced apoptosis. According to descriptions of it in many cell types and tissues, the HSPs induction is a well-conserved reaction to cigarette smoke exposure (Anbarasi et al., 2006a; Anbarasi et al., 2006b; Kreutmayer et al., 2011). Recent research has discovered that HSPA6 promotes growth and tumor progression in some cancer types and inhibits growth and progression in others. When the HSPA6 gene is overexpressed, it has an augmenting impact, preventing EJ cells from proliferating, migrating, and invading. By increasing the induction of the G2/M-phase-mediated ATM-CHK2-Cdc25C-p21WAF1-Cdc2 cascade, phosphorylating Akt and MAPK signaling, and suppressing the regulation of MMP-9 associated with transcription factor due to GE-induced changes in EJ cells, the enhancing impact of HSPA6 was demonstrated. Overall, innovative findings show that HSPA6 strengthens the inhibitory effects mediated by GE on EJ cell migration, invasion, and proliferation and can offer a fresh method for the management of malignant cancers (Zhang L. et al., 2023).

According to a study, HSPA6 is overexpressed in GC and encourages proliferation through the Hippo pathway. It is a promising therapeutic target and potential prognostic biomarker for GC. Between March 2015 and January 2017, 146 samples of paired stomach epithelial tissue from patients who had surgical excision at Xiamen University’s Zhongshan Hospital and Hospital of Jiangnan University were gathered. Twenty patients had freshly removed tissue that was immediately preserved in liquid nitrogen, and 146 patients had other samples that were formalin-fixed and paraffin-embedded. The integrated analysis of Coexpedia was used to examine the expression of HSPA6 in levels of mRNA from the samples of normal epithelial tissues of the stomach and GC samples (Zhang L. et al., 2023). When compared to healthy gastric epithelial tissues, the HSPA6 protein in GC tissues was noticeably upregulated. These results indicate that HSPA6 may function as a GC oncogene. The samples of GC were divided into the HSPA6high and HSPA6low groups using the cutoff score, which was 6, according to HSPA6’s score of IHC staining. The analysis of the Oncomine database showed that HSPA6 is overexpressed in breast cancer, CNS, and brain malignancies, cervical cancer, kidney cancer, lymphoma and leukemia, and GC and colorectal cancer. Compared to HSPA6low in the EGC group (P < 0.01), HSPA6high in the IGC group displayed substantially worse survival rates. Additionally, TNM stage and HSPA6 were found to be independent predictive variables for GC in univariate and multivariate Cox analyses (P < 0.05). The gene set enrichment analysis (GSEA) revealed that these genes’ signature activities were the cell cycle and the G2M checkpoint, respectively, demonstrating that HSPA6 is important for the growth of the cells of GC.

Multiple GC cell line transcriptome data were acquired from CCLE, and these data revealed that HSPA6 was downregulated in AGS cells, and it was significantly expressed in NUGC2 cells. According to GSEA results, the HSPA6-related genes were considerably enriched in the Hippo signaling network, which suggests that HSPA6 may encourage the proliferation of GC cells through the Hippo signaling route. The YAP and cyclin B1 expressions were dramatically upregulated, while p-YAP was significantly downregulated in the AGS cells when HSPA6 was overexpressed. In the NUGC2 cells, knocking down HSPA6 resulted in the opposite alterations. When combined, HSPA6 encourages GC cell proliferation by activating the Hippo pathway.

Through direct investigation of patient samples along with integrated transcriptome analysis from the databases UALCAN and GEPIA, it was found that in GC tissues, the expression of HSPA6 is abnormal relative to the stomach epithelium, which is normal. According to the patients’ classification based on *in situ* HSPA6 expression, overexpression of HSPA6 was substantially linked with larger tumors and IGC. A poor prognosis is predicted by high expression of HSPA6 in GC tissues, as shown by the fact that the overall survival (OS) of the HSPA6 high group was also lower than that of the HSPA6low individuals. The outcomes of the Cox analysis supported the notion that HSPA6 is an independent GC prognostic factor. A nomogram was developed for HSPA6 along with the clinical stage, with good prediction performance. These findings suggested that HSPA6 may serve as a prognostic indicator as well as an oncogene (Zhang L. et al., 2023).

#### 3.1.2 Stromal cell-derived factor 4

A study from Nagoya University identified SDF-4 as a promising biomarker for early detection of GC. Involving 396 GC patients and 80 healthy controls, the study showed that serum SDF-4 levels were significantly elevated even in stage I cases. It demonstrated high diagnostic accuracy with an AUC of 0.973, 89% sensitivity, and 99% specificity, far superior to traditional markers like CEA and CA19-9. Tissue analysis also confirmed consistent SDF-4 expression across tumor stages (Röcken, 2023).

#### 3.1.3 CAMSAP2

CAMSAP2 is a significant promoter of tumor progression in GC. Analysis of patient samples and public datasets revealed that CAMSAP2 is highly expressed in GC tissues and is associated with advanced tumor stage, higher grade, and elevated levels of serum markers such as CEA and CA19-9. Functional experiments showed that CAMSAP2 overexpression promotes epithelial–mesenchymal transition (EMT) by increasing mesenchymal markers (vimentin and N-cadherin) and reducing epithelial E-cadherin, increasing cancer cell migration and invasion. In contrast, silencing CAMSAP2 reversed these changes (Zuo et al., 2023).

#### 3.1.4 COL5A2, SPARC, COL1A1, and P4HA3

Researchers used a comprehensive integrative bioinformatic analysis approach using TCGA, GTEx, and Gene Expression Omnibus (GEO) datasets and found that COL1A1, COL5A2, SPARC, and P4HA3 were significantly upregulated in GC tissues compared to normal controls. These four genes, all associated with the extracellular matrix, were strongly correlated with poorer OS, DSS, and pathological stage. Specifically, ROC analyses revealed high diagnostic accuracy for GC: COL5A2 (AUC ≈ 0.802), SPARC (AUC ≈ 0.895), and P4HA3 (AUC ≈ 0.875) (Niu et al., 2022).

#### 3.1.5 AGTR1, DNER, EPHA7, and SUSD5

A 4-mRNA tissue-based panel comprising AGTR1, DNER, EPHA7, and SUSD5 was a powerful predictor of recurrence in locally advanced GC. The authors developed a risk stratification assessment model that combined these four markers with clinical features, achieving an AUC of 0.919 in the surgical cohort and 0.935 in an independent liquid biopsy validation cohort (Ding et al., 2024).

### 3.2 Urine proteomic signature

Urine collection is non-invasive and simple to do in large quantities compared to other biospecimens, making it possible to discover biomarkers during extensive population screening (Di Meo et al., 2017). Circulating urine molecules can potentially offer a comprehensive picture of a person’s health status. Malignant, apoptotic, and necrotic cells can release proteins into the blood, which the kidneys filter and reabsorb before the proteins eventually end up in the urine. Stable peptides and a few infrequent but crucial proteins can be tested with accuracy in urine (Gao, 2013; Jakobsen et al., 2015). Particularly, roughly 9% of the detected proteins in a healthy person’s urine are connected to the immune system (Marimuthu et al., 2011). These could be connected to the immune evasion and response seen in lesions before malignancy, and these were linked to significant somatic genomic changes (Krysan et al., 2019; William Jr et al., 2021). Changes in urine proteins could represent early biological processes involved in the development of GC carcinogenesis.

Finding biomarkers for the development of lesions in the stomach and for the possibility of developing early-stage GC may be made possible through the detection of proteins in urine. In one study by Fan H et al., proteome profiling in two stages among 255 patients with gastric lesions of various stages and GC using urine as a liquid biopsy was performed (Fan et al., 2022). Urine proteome signatures were created, and urine proteomic fingerprints were developed as they relate to the advancement of lesions of gastric mucosa and the possibility of developing GC. Forty-three proteins in urine were verified for the possibility of developing GC. A follow-up was conducted, and it was found that four proteins, namely, CDC42, SLC25A4, ANXA11, and NAPA, were strongly related to the probability of the progression of gastric mucosa lesions. GC tissue showed higher expression of ANXA11, CDC42, and NAPA than non-GC tissue did. All four proteins, namely, NAPA, ANXA11, SLC25A4, and CDC42, were present and enriched in the synaptic vesicle cycle (NAPA), cGMP-PKG signaling (SLC25A4), and endocytosis (CDC42) pathways. SLC25A4 was also shown in PPI network analysis as a key protein. A stratified analysis revealed consistently higher levels of these four proteins in progressed individuals, whether they were in the group with moderate gastric lesions at baseline (SG/CAG) or with advanced lesions (IM/LGIN) (Fan et al., 2022).

A study by Shimura et al. aimed to develop a non-invasive urinary protein biomarker panel for the early diagnosis of GC. Researchers analyzed urine samples from 372 individuals, dividing them into discovery, training, and validation cohorts. Using mass spectrometry and ELISA assays, they identified trefoil factor 1 (uTFF1) and ADAM12 (uADAM12) as key urinary biomarkers for GC, with *Helicobacter pylori* status included to improve diagnostic accuracy. In both training and validation cohorts, the combined panel (uTFF1, uADAM12, and *H. pylori* status) effectively distinguished GC patients from healthy controls, achieving area under the curve (AUC) values of 0.832 and 0.867, respectively. Sex-specific panels were also effective: uTFF1/uADAM12/*H. pylori* for men (AUC = 0.858) and uTFF1/uBARD1/*H. pylori* for women (AUC = 0.893). These panels showed high sensitivity even in stage I GC, with AUCs of 0.850 (males) and 0.845 (females), significantly outperforming conventional serum tumor markers. Importantly, urinary levels of the biomarkers were independent of *H. pylori* status, and the panels remained effective across GC stages. This study highlights the promise of a simple, cost-effective, and non-invasive urine test for early GC detection, potentially enabling mass screening and improving patient outcomes (Shimura et al., 2020).

Joshi and co-workers studied urinary proteomics to identify non-invasive biomarkers for early GC detection. Analyzing urine samples from GC patients and controls, the researchers identified six key proteins, ANXA11, CDC42, NAPA, LRG1, gelsolin, and CDH11, differentially expressed in GC. These biomarkers showed strong diagnostic performance and could distinguish GC patients from healthy individuals. Functional analysis indicated their involvement in cancer-related pathways such as cell adhesion and immune response. The findings suggest that urinary proteomics holds significant potential for precision oncology, offering a simple, non-invasive tool for early GC detection and disease monitoring, especially when combined with clinical parameters (Joshi et al., 2023).

Another study identified six urinary proteins, ANXA11, CDC42, NAPA, LRG1, gelsolin, and CDH11, as promising non-invasive biomarkers for GC detection (Husi et al., 2019). This study developed an XGBoost-based diagnostic model incorporating five plasma proteins, CDHR2, ICAM4, PTPRM, CDC27, and FLT1, achieving excellent discrimination for cardia GC (CGC) and its precursors. The model achieved AUCs of 0.931 for CGC, 0.867 for high-grade intraepithelial neoplasia (CHGD), and 0.763 for low-grade intraepithelial neoplasia (CLGD). Compared to traditional serological markers like PGII, PGR, and *H. pylori* status, the proteomic model demonstrated significantly better diagnostic accuracy (P for trend < 0.05), highlighting its potential as a non-invasive screening tool in high-risk populations (Li et al., 2024b).

#### 3.2.1 ANXA11

Annexins are a multigene family of phospholipid-binding proteins that are regulated by calcium (Ca2+) (Gerke and Moss, 2002; Gerke et al., 2005). These are linked to cell division, Ca2+ signaling, growth control, metastasis, apoptosis, cancer progression, and membrane traffic and organization (Moss and Morgan, 2004; Monastyrskaya et al., 2007). ANXA11 belongs to the family of proteins that bind phospholipids in a Ca2+-dependent manner, which includes calcyclin, an extended N-terminal domain with a binding site for another Ca2+-binding protein (CBP), and C-terminal repeated annexin (ANX) homology, which leads to Ca2+-dependent phospholipid binding (Lecona et al., 2003). Previous research has demonstrated that ANXA11 is linked to vesicle trafficking, signal transduction, cell differentiation, and apoptosis (Shibata et al., 2015; Hein et al., 2022). Ovarian, breast, liver, and colorectal cancers are significantly impacted by ANXA11, which is also linked to cancer recurrence, metastasis, medication resistance, and lymph node metastasis (Fernández-Madrid et al., 2004; Prieto-Fernández et al., 2022). It has been demonstrated that the downregulation of ANXA11 prevents the migration, growth, and invasion of the cells in GC, via the pathway of Akt/GSK-3 (Hua et al., 2018). Urinary ANXA11 levels showed a stepwise increase along the progression from non-atrophic gastritis to GC, indicating a strong association with malignant transformation. Importantly, elevated ANXA11 expression was also confirmed in GC tissue samples, suggesting concordance between urinary excretion and tumor expression. Multivariate logistic regression supported ANXA11 as an independent predictive marker for GC. This biomarker demonstrated potential in non-invasive screening, particularly for early-stage disease and mass surveillance in high-incidence regions. Baseline urinary ANXA11 levels were positively associated with progression from precancerous gastric lesions to malignancy (P < 0.05 via logistic regression).

In a recent study involving 63 paired GC and adjacent normal tissue samples, ANXA11 was found to be significantly upregulated at both the mRNA and protein levels in tumor tissues (Fan et al., 2021). High expression of ANXA11 was closely associated with adverse clinicopathological features, including larger tumor size, deeper invasion, lymph node metastasis, advanced TNM stage, and vascular invasion, suggesting a role in GC progression. Functional assays in GC cell lines (AGS and SGC-7901) revealed that silencing ANXA11 suppressed cell proliferation, colony formation, migration, and invasion, and caused cell cycle arrest in the G_0_/G_1_ phase. Mechanistically, these effects were mediated via downregulation of the Akt/GSK-3β signaling pathway, along with decreased expression of MMP-2, MMP-9, and cyclin D1. These findings indicate that ANXA11 functions as an oncogene in GC and may serve as a potential prognostic biomarker and therapeutic target. The limitation of this study was that it was conducted at a single center with a relatively small sample size of 63 paired tissue specimens, which may not adequately represent the diverse molecular characteristics of GC across different populations. Additionally, although the study demonstrated significant functional roles of ANXA11 *in vitro*, it lacked *in vivo* validation using animal models to confirm its effects on tumor growth and metastasis. The research also did not explore ANXA11’s potential as a prognostic marker, as it omitted survival analyses such as Kaplan–Meier curves or correlations with patient outcomes. Mechanistically, the study identified involvement of the Akt/GSK-3β signaling pathway but did not delve deeply into upstream regulators or downstream targets of ANXA11. Lastly, ANXA11 was evaluated only in tumor tissues, without examining its expression or utility as a non-invasive biomarker in serum or urine, limiting its clinical applicability in early detection or monitoring (Hua et al., 2018).

#### 3.2.2 CDC42

The human homolog of *Saccharomyces cerevisiae* CDC42 (cell division control protein 42) was identified in 1990. CDC42 is a member of the GTPase Rho family. The attached nucleotide controls CDC42’s activity, and three different sets of regulatory proteins regulate these in turn. Guanine nucleotide exchange factors (GEFs), which enable the conversion of GDP to GTP, regulate the activation of Cdc42 (Hodge and Ridley, 2016). It is generally known that because of the crucial physiological roles that Cdc42 performs, it plays a significant part in malignancies. Cdc42 plays a role in the development of tumors as well as invasion and metastasis by controlling cell cycle progression, vesicle trafficking, microtubule and cytoskeletal dynamics, phagocytosis, apoptosis, cell polarity, and transcription. Inhibiting CDC42 and its signaling pathway elements is an appealing therapeutic target. These targets are currently being investigated with small molecules and biologics (Murphy et al., 2021). The invasion and migration of cells in GC were dramatically inhibited by CDC42 gene knockdown. In an *in vitro* study, CDC42 knockdown in AGS and SGC-7901 GC cells led to G_1_/S cell cycle arrest and significantly reduced cell proliferation, migration, and invasion. It also downregulated key proteins, including cyclin A, cyclin D1, cyclin E, PCNA, and MMP-9, confirming CDC42’s role in promoting GC progression (Du et al., 2016).

In a 2021 study published in Molecular Therapy Nucleic Acids, researchers demonstrated that miR-497 acts as a tumor suppressor in GC by directly targeting CDC42. miR-497 expression was significantly downregulated in GC tissues and cell lines. Overexpression of miR-497 in HGC-27 cells reduced proliferation by approximately 40%, inhibited migration and invasion by over 50%, and suppressed EMT, as evidenced by increased E-cadherin and decreased N-cadherin and vimentin levels. *In vivo*, mice injected with miR-497–transfected cells showed a ∼60% reduction in liver metastasis nodules compared to controls. Luciferase reporter assays confirmed CDC42 as a direct target of miR-497, and co-transfection with CDC42 restored cell proliferation and metastatic potential, reversing the effects of miR-497. These results confirm the functional importance of the miR-497/CDC42 axis in regulating GC progression and suggest its potential as a therapeutic target for inhibiting tumor metastasis (Zhang L. et al., 2021).

Mechanistically, miR-148b-3p inhibited the Dock6/Rac1/CDC42 signaling axis, leading to reduced activation of downstream effectors involved in cytoskeletal reorganization and metastasis. In a mouse tail vein injection model, miR-148b-3p overexpression reduced lung metastasis nodules by approximately 70%. Clinically, analysis of 92 GC tissue samples showed that low miR-148b-3p expression and high Dock6 expression were significantly correlated with lymph node metastasis, advanced TNM stage, and poor overall survival (P < 0.01). These findings establish the miR-148b-3p/Dock6/Rac1/CDC42 axis as a critical regulator of GC metastasis and a potential therapeutic target (Li X. et al., 2018).

In a study on MICAL2’s role in GC cell migration, Cdc42 emerged as a critical mediator. Researchers found that knockdown of MICAL2 significantly reduced the levels of active (GTP-bound) Cdc42, while MICAL2 overexpression had the opposite effect. Constitutively active Cdc42 (Q61L mutant) restored the migratory ability and reversed the upregulation of E-cadherin induced by MICAL2 depletion, confirming that Cdc42 activation is essential for MICAL2-driven cell migration and E-cadherin degradation. These findings highlight Cdc42 as a key downstream effector of MICAL2 that drives cytoskeletal remodeling and metastatic behavior in GC (Wang et al., 2022).

In a 2019 study published in theranostics, researchers demonstrated that GINS4 promotes GC progression by directly activating CDC42. GINS4 expression was significantly higher in GC tissues compared to normal tissues, and its overexpression correlated with poor differentiation, advanced TNM stage, deep invasion, and lymph node metastasis (P < 0.01). Mechanistically, GINS4 was found to physically bind to CDC42, as confirmed by GST pull-down and co-immunoprecipitation assays. Overexpression of GINS4 increased GTP-bound (active) CDC42 levels by ∼2.3-fold, whereas GINS4 knockdown reduced CDC42 activity by ∼60%. This activation led to increased PI3K/Akt and MAPK/ERK pathway signaling, along with increased expression of cyclin D1, Ki-67, N-cadherin, and vimentin, and decreased E-cadherin, supporting EMT progression. Functionally, GINS4 overexpression resulted in a ∼1.9-fold increase in cell proliferation, >2-fold increase in migration and invasion, and significantly larger tumor volume in nude mice xenografts (mean tumor volume: 740 mm^3^ vs. 370 mm^3^; P < 0.01). These results establish CDC42 as a direct and critical downstream effector of GINS4, mediating its pro-tumorigenic effects in GC (Zhu et al., 2019).

While these studies provide important insights into the role of CDC42 in GC, several limitations should be noted. Most were conducted with relatively small sample sizes and lacked validation in large or multicenter clinical cohorts, which limits the generalizability of their findings. The conclusions were primarily based on *in vitro* experiments and xenograft models, which do not fully mimic the complexity of human tumors and the tumor microenvironment. Additionally, while the studies identified CDC42 as a key effector in various oncogenic pathways, they focused narrowly on single regulatory axes and did not explore broader signaling networks or compensatory mechanisms. None of the studies assessed pharmacological inhibition of CDC42, reducing their immediate translational relevance. Furthermore, genetic diversity, patient heterogeneity, and long-term clinical outcomes were not adequately addressed. These limitations suggest that more comprehensive clinical and mechanistic studies are needed to validate CDC42 as a reliable biomarker and therapeutic target in GC.

#### 3.2.3 SLC25A4

SLC25A4 is an ATP/ADP transporter that controls mitochondrial energy production by ensuring that the delicate equilibrium exists between thermogenesis and ATP synthesis (Namba et al., 2020). SLC25A4 upregulation reflects mitochondrial malfunction, which could be a key factor in the development of cancer (Moreno-Sánchez et al., 2007; Clémençon et al., 2013). SLC25A4 is a component of the cGMP/PKG pathway, whose activation is essential for GC cell proliferation, metastasis, and chemoresistance (Xiang et al., 2021). In a study by Fan et al. (2022), large-scale urine proteomics of high-risk individuals identified 246 differentially expressed proteins, with four proteins, SLC25A4, CDC42, NAPA, and ANXA11, showing strong associations with GC development. Among these, CDC42, NAPA, and ANXA11 were significantly elevated in both urine and matched tissue samples of GC patients. CDC42 showed a fold change of 1.98 (P < 0.01), NAPA had a fold change of 2.10 (P < 0.01), and ANXA11 showed a fold change of 1.85 (P < 0.01) in tissue compared to normal controls. These findings support their role as intracellular biomarkers linked to cytoskeletal regulation, membrane trafficking, and nucleoplasmic signaling during GC progression.

SLC25A4, a mitochondrial ADP/ATP translocator and component of the cGMP/PKG pathway, was significantly upregulated in urine (fold change 1.67, P < 0.05), suggesting mitochondrial dysfunction as an early systemic feature of malignancy. However, SLC25A4 did not show significant upregulation at the tissue level, indicating that its role may be more reflective of systemic metabolic stress than localized tumor activity. To assess clinical utility, a survival analysis of GC patients was performed using tissue data. It revealed that high expression levels of FAP (HR: 2.12, P = 0.004), PSAPL1 (HR: 1.91, P = 0.011), and SERPINH1 (HR: 2.45, P = 0.001) were significantly associated with poor overall survival. As a result, these three proteins were proposed as prognostic biomarkers for GC, while CDC42, NAPA, and ANXA11 remained leading candidates for early-stage diagnosis based on their consistent expression patterns in both urine and tissue (Fan et al., 2022).

### 3.3 Differentially expressed genes: FAP, SERPINH1, and PSAPL1

DEGs represent genes whose expression levels are significantly altered between cancerous and non-cancerous tissues and are key indicators of the molecular changes underlying GC pathogenesis (Liang et al., 2021; Abdolahi et al., 2023). These gene expression alterations often reflect oncogenic signaling, tumor–stromal interactions, immune modulation, or metabolic reprogramming. As such, DEGs serve as a valuable source for identifying potential diagnostic and prognostic biomarkers (Wang et al., 2015). In GC, numerous DEGs have been identified through transcriptomic profiling, and several have shown consistent dysregulation across independent datasets. Notably, genes such as FAP, SERPINH1, and PSAPL1 have emerged as promising biomarker candidates due to their significant upregulation in GC tissues and their functional relevance in tumor progression, extracellular matrix remodeling, and immune evasion. The number of diagnostic biomarkers related to GC was initially found using machine learning in the Gene Expression Omnibus dataset, and they were then verified in the Cancer Genome Atlas. The upstream regulatory lncRNA, along with miRNA, was then forecasted in reverse and confirmed from the standpoint of prognostic value and expression pattern using a variety of bioinformatic approaches. In the end, a new ceRNA regulatory network was effectively created, and every element of the network completely complies with the prognosis made by the ceRNA theory while also being associated with the prognosis of patients having GC.

The algorithms SVM-RFE and LASSO were used to find potential biomarkers from these DEGs. Results showed 40 DEGs that act as possible GC biomarkers based on the SVM-RFE approach, while 13 DEGs were identified as such based on the LASSO algorithm. The Venn diagram was used to identify six possible biomarkers that overlapped, which included MT1M, FAP, ADH7, SERPINH1, CDH3, and PSAPL1. Additionally, the training cohort’s ROC curves were used to determine the diagnostic effectiveness of these prospective biomarkers. Important GC indicators include MT1M, FAP, ADH7, SERPINH1, CDH3, and PSAPL1, according to the findings, which showed that the AUCs of all of these putative biomarkers were more than 0.9. Finally, survival analysis was carried out to check the predictive usefulness of these putative biomarkers. It revealed a strong correlation between GC patients’ prognosis and deregulation of SERPINH1, FAP, and PSAPL1. Thus, SERPINH1, FAP, and PSAPL1 were identified as potential GC biomarkers (Li et al., 2022).

#### 3.3.1 FAP

Among the dipeptidyl peptidase (DPP)-IV activity and/or structure homologs (DASH), FAP (familial adenomatous polyposis) is a non-classical serine protease (Šedo and MalíK, 2001; Bušek et al., 2004). The FAP gene is extremely conserved in many species. In both humans and mice, it is confined to the long arm of the chromosome (Tillmanns et al., 2024). FAP is situated next to DPP-IV/CD26, which is FAP’s nearest homologue and, like DPP-IV, it has 26 exons. FAP is consequently believed to have developed by the duplication of genes. The single-pass 760 amino acid type 2 transmembrane protein that the human FAP gene encodes is made up of a long extracellular domain, a brief cytoplasmic N-terminal section, and a transmembrane region (amino acids 7–26) (Busek et al., 2018).

FAP’s soluble form is found in the plasma of blood of different species, although most normal adult tissues express FAP insignificantly or not at all (Lee et al., 2006; Keane et al., 2014). F19 murine monoclonal antibody produces FAP, which was first discovered to be an antigen against lung fibroblasts that could be detected. The antigen has been demonstrated to be a tissue-level feature of the stroma in a number of cancers, although its expression under healthy circumstances is highly restricted (Busek et al., 2018). Further research has increased the number of cancers where FAP is overexpressed and shown that FAP can be found in various elements of the cell microenvironment of the tumor, in addition to cancer-associated fibroblasts. At the invasion front of GC, increased tissue FAP is linked to poorer prognosis, lower tumor cell differentiation, higher TNM stage, and serosal invasion (Shan et al., 2012). Poorer survival is linked to a higher stromal FAP (Wen et al., 2017). In intestinal-type GC, FAP is expressed more strongly in the endothelial cells, stroma, and moderately differentiated cancer cells compared to GC of diffuse type (mostly in endothelial cells, cells of cancer with limited cellular connections). The presence of liver and lymph node metastases is linked to a higher stromal FAP expression in intestinal-type GC (Okada et al., 2003).

Comparing GC tissues to normal controls, it was shown that FAP was significantly expressed in GC tissues, and patients in higher GC stages have an increased level of FAP compared to patients with early-stage GC (Gao et al., 2019). Additionally, research revealed that GC cells overexpressed FAP and that knocking down FAP greatly reduced GC cell invasion and migration by inhibiting CAF activity (Wang et al., 2013). In a study of 171 GC patients receiving neoadjuvant chemotherapy (NCT), FAP-positive CAFs were significantly associated with poor tumor regression grade (TRG) (P < 0.01) and worse overall survival in univariate analysis (P < 0.05). High post-treatment FAP expression remained an independent predictor of poor TRG in multivariate logistic regression (P < 0.010). Elevated FAP levels also correlated with advanced ypT/ypN/ypTNM stages, poor differentiation, and increased lymphovascular and perineural invasion. FAP+ CAFs were strongly linked with expression of EMT markers (e.g., Twist1, P = 0.001) and CSC markers (CD44, ALDH1, LGR5; P < 0.05), indicating their role in chemoresistance. However, in multivariate Cox analysis, FAP was not an independent predictor of overall survival, highlighting its value primarily as a marker of treatment response rather than long-term prognosis (Zhao and Zhu, 2023).

Cai et al. performed a multifactorial analysis to explore the role of FAP in gastrointestinal cancers, including gastric (STAD), colorectal, pancreatic, and liver cancers. FAP was found to be significantly overexpressed in tumor tissues across all cancer types studied (P < 0.001), particularly within cancer-associated fibroblasts. Its expression correlated strongly with extracellular matrix-related genes such as COL1A1, COL3A1, and POSTN, and with increased infiltration of immunosuppressive M2 macrophages (Cai et al., 2023). In a joint bioinformatics analysis using the TCGA and GEO datasets, FAP was identified as a key diagnostic and prognostic biomarker in GC. FAP was significantly overexpressed in tumor tissues compared to normal tissues (P < 0.001), with expression levels increasing alongside clinical stage and T-stage (P < 0.05). High FAP expression was associated with shorter overall and disease-specific survival, and multivariate Cox regression confirmed it as an independent risk factor for overall survival (HR = 1.56, 95% CI: 1.12–2.17, P = 0.008). Diagnostic ROC analysis yielded an AUC of 0.870, indicating high accuracy in distinguishing cancer from normal tissue. Moreover, FAP expression positively correlated with immune infiltration, particularly CD4^+^ T cells, macrophages, and CAFs, while showing a negative correlation with tumor purity (Zhang K. et al., 2023).

A recent study identified FAP^+^ GCMSCs as a dominant stromal subset driving GC progression. Using single-cell RNA sequencing, clinical pathology, and functional assays, FAP^+^ GCMSCs were shown to promote GC cell proliferation, migration, and stemness via paracrine INHBA secretion and SMAD2/3 activation. Additionally, they increased ECM collagen deposition, which interacted with ITGB1, activating FAK–YAP signaling (Liu et al., 2025).

#### 3.3.2 SERPINH1

The precise secretion and folding of various types of collagen depend on the collagen-specific protein called SERPINH1, also known as HSP47 (Duarte and Bonatto, 2018). Cervical cancer (Yamamoto et al., 2013), breast cancer (Lai et al., 2017; Zhu et al., 2015), glioblastoma (Zhao et al., 2014), and colorectal cancer (Mori et al., 2017) all have abnormal expression of SERPINH1. SERPINH1 encourages breast cancer cell metastasis and invasion by influencing the levels of various extracellular matrix (ECM) proteins (Lai et al., 2017). In GC, most of the genes are implicated in the EMT pathway, according to gene enrichment analysis. Additionally, GC EMT is regulated by the Wnt/-catenin signaling system (Gao et al., 2022). Compared to normal gastric mucosal tissues, GC tissues have considerably higher levels of mRNA of SERPINH1. In GC patients, SERPINH1 is a prognostic marker (Tian et al., 2020). An increased level of SERPINH1 was also detected in squamous cell carcinoma of the esophagus (Lee et al., 2016), cervical squamous cell carcinoma (Yamamoto et al., 2013), and ulcerative colitis-associated carcinomas (Mori et al., 2017). The protein SERPINH1 is localized in the ECM of scirrhus GC tissues and cells (Hirai et al., 2006). GC tissues exhibit more SERPINH1 protein than do typical gastric mucosal tissues (Tian et al., 2020). According to studies by Kawagoe et al. (2020) and Tian et al. (2020), GC tissues have much higher levels of SERPINH1 protein and mRNA expression than normal tissues, and inhibiting SERPINH1 dramatically reduced the potential of cancer cells to migrate and invade (Kawagoe et al., 2020; Tian et al., 2020). Additionally, it is reported that increased SERPINH1 levels are linked to GC patients’ poor prognosis (Tian et al., 2020).

A study identified SERPINH1 as a novel prognostic biomarker in GC. Through comprehensive bioinformatics analysis using three GEO datasets, 360 DEGs were identified, among which SERPINH1 emerged as a key gene. It was significantly overexpressed (log FC = 3.6, P = 2.36 × 10^−18^) and correlated with poor survival (P < 0.001). Functional assays showed reduced proliferation and migration upon SERPINH1 knockdown (Qiu et al., 2023). In a study by Li et al., potential biomarkers were screened and validated by various machine learning approaches. The authors found 188 DEGs, and three biomarkers, FAP, PSAPL1, and SERPINH1, were identified. H19 and miR-378a-5p bind with SERPINH1 in GC. It was also found as an upstream regulatory IncRNA that regulates the SERPINH1 in GC (Li et al., 2022).

#### 3.3.3 PSAPL1

A study identified PSAPL1 as one of three candidate diagnostic biomarkers for GC, along with FAP and SERPINH1. The study found that PSAPL1 is part of a ceRNA network involving H19 and miR-378a-5p, which is correlated with immune cell infiltration. Further analysis of this ceRNA network revealed that miR-378a-5p is associated with a favorable prognosis in GC patients. PSAPL1 is also linked to pathways involved in extracellular matrix organization and cell adhesion, which are important in tumor progression. Using machine learning methods (LASSO and SVM-RFE), the authors identified PSAPL1 as one of six overlapping candidate diagnostic genes (including ADH7, CDH3, FAP, MT1M, PSAPL1, and SERPINH1). Notably, it achieved an impressive diagnostic accuracy in TCGA GC cohorts (AUC = 0.731) (Li et al., 2022). Dysregulated expression of PSAPL1 was also significantly associated with patient prognosis in TCGA stomach adenocarcinoma cohorts (Wen et al., 2022). In integrated transcriptome comparisons, PSAPL1 emerged as one of 27 genes significantly dysregulated in both TCGA-STAD and patient-specific sequencing data; notably, high PSAPL1 expression was associated with poorer overall and disease-free survival (log-rank P < 0.05) (Hu et al., 2020). However, in survival analysis using the KM-Plotter database—covering 197 stage III GC patients—PSAPL1 expression did not reach statistical significance in predicting overall survival (log-rank P = 0.061) (Sarma et al., 2022).

### 3.4 Salivary biomarkers

One of the body’s most intricate biological fluids, saliva reflects a variety of physiological states (Kaczor-Urbanowicz et al., 2017; Pedersen et al., 2018). Compared to biopsy or blood sampling, saliva collection and storage are convenient, less invasive, less expensive, and require no specialized equipment (Zhang et al., 2012). Salivary biomarkers can reflect both local and systemic pathological changes, and recent advances in proteomic and transcriptomic technologies have enabled the identification of specific proteins and microRNAs (miRNAs) in saliva that are differentially expressed in GC patients (Bonne and Wong, 2012; Wang X. et al., 2017). Among the protein-based candidates, cystatin B, a cysteine protease inhibitor involved in inflammatory responses and proteolytic balance, has been found to be upregulated in the saliva of GC patients. DMBT1 is a multifunctional protein associated with mucosal immunity and epithelial integrity, is significantly altered in GC saliva samples, and has been linked to tumor-related immune responses. Another protein, TPI1, is a glycolytic enzyme that has been reported as a salivary biomarker reflecting metabolic reprogramming in gastric tumors. In addition to proteins, salivary miRNAs have shown strong promise due to their stability and diagnostic specificity. miR-376c and miR-140-5p are two such salivary miRNAs that have been consistently dysregulated in GC patients. These miRNAs are implicated in tumor growth, metastasis, and apoptosis regulation and may serve as early indicators of malignancy. Salivary proteins have been employed in many investigations as possible diagnostic indicators and to observe treatment, disease prognosis, and patient survival (Shu et al., 2017; Rapado-González et al., 2021).

#### 3.4.1 Cystatin B

An intracellular thiol protease inhibitor known as cystatin B (CSTB), encoded by the CSTB gene, is a protein (Ma Y. et al., 2017). Chromosome 21q22.3 is the location where the CSTB gene is located (Joensuu et al., 2008). This protein is a member of the second and vast type of cystatins, can inhibit cathepsins B, L, H, and papain, and can form noncovalent force-stabilized dimers. It is believed that this protein helps prevent protease leakage from lysozymes (Zhang et al., 2016; Soond et al., 2019). It was discovered that GC patients had considerably lower mean CSTB levels in saliva than controls (P = 0.001). According to the Spearman test of correlation, in controls, there is a notable positive correlation between levels of CSTB in saliva and age. However, there was no statistically significant correlation between these two parameters. It was revealed through the outcomes of multiple linear regression that decreased CSTB levels in saliva are notably linked to GC, after controlling for the effects of participant age. With a one-unit increase in levels of CSTB in saliva, there is a 7% abatement in GC risk. To estimate salivary CSTB diagnostic values, a ROC curve was also constructed. Salivary CSTB levels of 119.06 ng/mL or less were the ideal ceiling point to distinguish controls from patients having GC. At this cutoff value, the specificity was 70.97%, and the sensitivity was 83.87% (Koopaie et al., 2022).

The PI3K/Akt/mTOR pathway is suppressed by the overexpression of CSTB. The PI3K/Akt/mTOR pathway regulates numerous cellular activities like cell proliferation, angiogenesis, and metabolism (Zhang et al., 2016; Qiu et al., 2020). By influencing cell migration and proliferation, downregulation of CSTB occurs, which leads to GC progression and development. Earlier research demonstrated several roles of CSTB in colon cancer, ovarian cancer (Ma Y. et al., 2017), and myoclonus (Di Matteo et al., 2020). According to the study, GC patients’ salivary CSTB levels were lower than those of the control group. Additionally, this biomarker distinguished GC from the healthy control with a specificity of 70.97% and a sensitivity of 83.87% (Koopaie et al., 2022). Previous research has demonstrated that CSTB can be used as a diagnostic marker for GC by downregulating GC protein and mRNA levels (Giganti et al., 2019). Recently, Xu et al. identified CSTB as a gene related to the prognosis and altered apoptosis in GC cells (Xu et al., 2024).

#### 3.4.2 Deleted in malignant brain tumor 1 (DMBT1)

Deleted in malignant brain tumor 1 (DMBT1) is a tumor-inhibiting gene that is inactivated in numerous medulloblastoma cell lines compared to normal cells and is situated on chromosome 10q25.3-q26.1 (Madsen et al., 2013; Brim and Ashktorab, 2016; Shen et al., 2016; Garay et al., 2017). It plays a crucial part in some biological responses, including the innate immune system, accumulation and recognition of bacteria, and inflammation, when it binds to different host molecules and pathogens. DMBT1 may lead to polarization of epithelial cells by functioning as a factor for the differentiation of the epithelium. The protein DMBT1, a member of the scavenger receptor cysteine-rich (SRCR) family, is produced by the DMBT1 gene (Garay et al., 2017; Park H. S. et al., 2018).

DMBT1 is a multifunctional protein that mediates interactions between cells and the extracellular matrix and is associated with the mucins and Mac-2 binding proteins (Mollenhauer et al., 2003; Roldán and Marini, 2014). The DMBT1 variants DMBT1GP340 (glycoprotein-340) and DMBT1SAG (salivary agglutinin), related to epithelial regeneration and innate host defense, are present in both the oral cavity and the respiratory tract (Mollenhauer et al., 2000; Prakobphol et al., 2000; Kang and Reid, 2003). Due to its capacity to promote migration of alveolar macrophages and its interaction with lectins surfactant protein A and D (Sp-a, Sp-D), DMBT1GP340 participates in innate host defense in the respiratory tract (Braidotti et al., 2004; Li J. et al., 2017). The rabbit homolog of DMBT1, which is Hensin, promotes cell–extracellular matrix (ECM) interactions that lead to epithelial terminal differentiation, and this may also be true of DMBT1 (Mollenhauer et al., 2000; Roldán and Marini, 2014). Decreased levels of DMBT1 promote the formation of tumor cells, whereas when it is translocated to the ECM, it initiates cellular differentiation (Mollenhauer et al., 2002). The levels of DMBT1 and cellular location in hyperplastic, normal, and neoplastic cells of the breast epithelium differ. It is upregulated and polarized toward the glandular lumen or the basolateral membrane in normal or hyperplastic tissue around carcinomas, but it is downregulated in carcinomas. Furthermore, it was established that MCM5 and DMBT1 are co-expressed in both healthy and hyperplastic tissue. It was proposed that in these epithelia, the DMBT1’s basolateral relocation and its hyperexpression inhibit the uncontrolled development that characterizes neoplastic epithelium (Braidotti et al., 2004).

The expression of DMBT1 in various malignancies has fluctuated, according to several studies reporting conflicting results (Zhang, 2019; Gan et al., 2021). DMBT1 is eliminated or diminished in a number of tumor types, according to preliminary investigations (Hoki et al., 2019). In individuals having atrophic gastric mucosa and gastric mucosal dysplasia, the levels of DMBT1 significantly rise (2.5-fold) in the mucosa (Garay et al., 2017). *H. pylori* infection was linked to an increase in advanced gastritis. Additionally, precancerous lesions of the gastric mucosa were shown to have elevated expression of DMBT1, and DMBT1 played a complex role in the development of gastric carcinogenesis (Sousa et al., 2012; Garay et al., 2017). According to research by Conde et al., DMBT1 upregulates mRNA in 62% and downregulates it in 38% of GC patients. Differentiated GCs are more likely to lose DMBT1 while all types of GCs are to upregulate it (Conde et al., 2007). When GC patients were compared to healthy controls, the mean DMBT1 concentration was considerably greater (P = 0.002). The DMBT1 levels and participant ages were not significantly correlated in either group (P = 0.07 and r = 0.33 in the case of patients; P = 0.13 and r = 0.28 in the case of the control group). There was no correlation between any other explanatory variables and the concentration of DMBT1 in either group. After taking into account the effects of the participants’ ages, the analysis of multiple linear regression showed that GC was connected with DMBT1 increased levels (adjusted P = 0.001, F = 8.67, and R2 = 0.20). The levels of DMBT1 and CSTB in saliva showed no correlation between the control group and GC patients (P = 0.85 and r = 0.04) and healthy controls (P = 0.33 and r = 0.18). An ROC curve was constructed to differentiate the control group from GC patients to evaluate diagnostic values. The findings revealed that the ROC curve’s area was found to be 0.741 (95% CI = 0.614 to 0.844; P < 0.001). A level of DMBT higher than 146.33 ng/mL was the ideal cutoff value for differentiating the control group from GC patients, with specificity and sensitivity of 64.52% and 80.65%, respectively.

Salivary DMBT1 was proposed as a non-invasive marker for detecting GC due to its adequate sensitivity and specificity in GC detection (Koopaie et al., 2022). In a recent study, cells treated with both *H. pylori* and si-DMBT1 showed the highest growth, migration, and invasion. DMBT1 mRNA and protein levels were lowest in this group. Overall, *H. pylori* appears to promote GC progression by suppressing DMBT1, linking infection and inflammation to tumor development (Zhou et al., 2025).

#### 3.4.3 Triosephosphate isomerase (TPI1)

Xiao et al. (2016) conducted a quantitative proteomics study using TMT labeling on salivary samples from 40 GC patients and 40 matched controls. Among over 500 quantified proteins, 48 showed significant differential expression; five were selected for ELISA validation. TPI1, along with CSTB and DMBT1, was confirmed as significantly altered in GC patients (P < 0.05). As a panel of all three, these biomarkers achieved ∼85% sensitivity, ∼80% specificity, and an accuracy of 0.93 in detecting GC (Xiao et al., 2016). A systematic review registered under PROSPERO (CRD42021259519) reaffirmed that among salivary biomarkers for GC, the trio CSTB, TPI1, and DMBT1 emerged with the highest diagnostic accuracy in preclinical validation stages (Zúñiga-Pérez et al., 2024).

#### 3.4.4 miR-376c

In a study by Machida et al. (2015), salivary exosomes from GC patients and healthy individuals were profiled using microarray and qRT-PCR. miR-376c was significantly upregulated in GC patients. The study showed that miR-376c could distinguish GC patients from healthy controls with an AUC of 0.82, a sensitivity of 78.3%, and a specificity of 83.3%. Exosomal miR-376c originates from tumor cells and reflects active tumor signaling pathways, particularly those associated with proliferation and metastasis (Machida et al., 2015). Song et al. conducted a nested case-control study using serum samples from a large Chinese prospective cohort to evaluate microRNAs as early detection tools for GC. The authors identified a panel consisting of miR-376c, miR-221, and miR-744, all of which were significantly upregulated in the serum of GC patients compared to controls. Notably, this panel could detect GC up to 5 years prior to clinical diagnosis, suggesting strong potential for early screening. The 3-miRNA panel achieved a sensitivity of 82.4% and a specificity of 58.8%, offering a useful tool for risk stratification (Song et al., 2012).

#### 3.4.5 miR-140-5p

miR-140-5p is significantly downregulated in GC tissues and functions as a tumor suppressor by directly targeting the YES1 oncogene. Quantitative RT-PCR analyses and *in situ* hybridization across GC patient samples confirmed the inverse relationship between miR-140-5p and YES1 expression. Functional assays in AGS and BGC-823 cell lines showed that overexpression of miR-140-5p significantly inhibited cell proliferation, migration, and invasion, while luciferase reporter assays validated YES1 as a direct target. In xenograft mouse models, miR-140-5p overexpression markedly reduced tumor growth, underscoring its therapeutic potential (Fang et al., 2017). The interplay between long non-coding RNA SNHG20, miR-140-5p, and NDRG3 revealed a regulatory axis impacting 5-fluorouracil resistance in GC. They discovered that miR-140-5p directly targets NDRG3, a gene overexpressed in GC, and downregulates its expression (Yu et al., 2019).

### 3.5 lncRNA biomarkers

Long non-coding RNAs (lncRNAs) are a class of non-protein-coding transcripts longer than 200 nucleotides that play crucial regulatory roles in gene expression at transcriptional, post-transcriptional, and epigenetic levels. In recent years, lncRNAs have emerged as key players in cancer biology, including GC, where they influence various processes such as proliferation, apoptosis, invasion, and metastasis. Due to their cancer-specific expression patterns and high stability, lncRNAs are increasingly being explored as promising biomarkers for early detection and prognosis in GC. One of the most extensively studied lncRNAs in this context is NEAT1 (nuclear paraspeckle assembly transcript 1), which has been reported to be significantly upregulated in GC tissues and associated with tumor growth, epithelial–mesenchymal transition (EMT), and chemoresistance. In addition, N6-methyladenosine (m6A)-related lncRNAs, which are regulated through RNA methylation modifications, have gained attention for their involvement in cancer-related signaling pathways and immune regulation.

#### 3.5.1 NEAT1

NEAT1, one of the several types of lncRNAs that are now understood, resides on chromosome 11 and has five recognized splice structures. According to the Gene Cards, this gene produces a long non-coding RNA (lncRNA) that has been deciphered from a variety of endocrine neoplasia loci. The lncRNA frames the central portion of sub-organelles of the paraspeckle by remaining in the core. For a very long time, NEAT1 can also function as a controller of transcription while incorporating some characteristics linked to the spread of disease. NEAT1 can facilitate both the RISC complex and miRNA binding. This lncRNA plays a critical part in the positive regulation of inflammatory response, the negative regulation of miRNA-mediated gene silencing, and the positive regulation of synoviocyte proliferation (Zhang Y. et al., 2017).

NEAT1’s promoter region mutations have been linked to the development of cancer in normal breast and renal cells (Li S. et al., 2018; Rheinbay et al., 2020). Recent research has shown the role of NEAT1 in the development of a variety of types of cancer. As an example, the lncRNA NEAT1 was shown to have remarkably high levels in the cells and tissues of colorectal cancer (CRC). They also showed that knockdown of NEAT1 can encourage CRC cell invasion and apoptosis and can sponge a new microRNA (miR-150-5P) (Wang et al., 2020b). Gao M et al. showed the development of GC via controlling ABCC4 and miR-356a-3p sponging because of NEAT1 (Gao et al., 2020). Transcription 3 activator and signal transducer may regulate the expression of NEAT1, and downstream gene transcription is affected by the altered expression of NEAT1 epigenetically during Alzheimer’s disease and HSV-1 (herpes simplex virus-1), suggesting the function of NEAT1 as an effector and stress sensor, in addition to the target-genes expression, which is influenced by the molecular mechanism of NEAT (Wang Z. et al., 2020). In many different kinds of human cancer, NEAT1 is the nuclear-enriched abundant transcript 1 (NEAT1) lncRNA (Dong et al., 2018). A worse prognosis for cancer patients is highly correlated with increased NEAT1 expression (Lanzós et al., 2017; Dong et al., 2018).

The clinical characteristics of cancer that NEAT1 is responsible for include recurrence, patient survival, stem cell-like phenotype, and metastasis (Lanzós et al., 2017). The malignant behavior of tumor cells in breast cancer (Li W. et al., 2017; Zhao et al., 2017), hepatocellular carcinoma (Fujimoto et al., 2016), laryngeal squamous cell carcinoma (Wang et al., 2016), lung cancer (Sun et al., 2016), glioma (Zhen et al., 2016), prostate cancer (Chakravarty et al., 2014), and skin cancer (Adriaens et al., 2016) can be reduced by NEAT1 knockdown. It has been determined that NEAT1 mediates transcription (Hirose et al., 2014; West et al., 2014) and is an essential component of nuclear paraspeckles (Clemson et al., 2009). A novel target for cancer therapy and cancer detection, ncRNA m6A played an important role in controlling prostate cancer progression (Wen et al., 2020).

NEAT1 promoted tumor growth and metastasis through the action of DDX5. In contrast to healthy tissues, malignant tissues have an overexpression of DDX5, which is found in the cell nucleus. In 71 CRC patients, NEAT1 expression correlated positively with DDX5 expression. NEAT1 is crucial to the advancement of CRC, and by directly binding to DDX5, it stimulated-catenin transcriptional activity, which may be indicative of the underlying molecular pathways driving their biological functions. These findings offer novel perspectives on the functions of lncRNAs in the development of CRC (Zhang M. et al., 2018). Nuclear architectural lncRNA NEAT1 is very prevalent. NEAT-1 and NEAT-2, the two NEAT isoforms, overlap, with the latter acting as a scaffold in the production of a type of nuclear ribonucleoprotein body (paraspeckles) (Knutsen et al., 2022). It has recently been discovered that NEAT-2 expression predicts progression-free survival in patients with ovarian cancer receiving platinum-based therapy but not NEAT-1 (Adriaens et al., 2016).

The level of NEAT1-2 is notably correlated with HER2 in samples of breast cancer and with the grade of breast cancer. During lactation in human breast tissue, NEAT-2 expression is also elevated (Knutsen et al., 2022). A study by Azadeh et al. (2022) revealed differences in NEAT-1 levels between samples of breast cancer and GC from the database of gastric and breast cancer, which is high throughout the Isfahan community. According to data analysis and the ENCORI, NEAT1 is significantly downregulated in cases of GC. The GC samples displayed a considerable downregulation of NEAT1, according to the analysis of GEPIA2 online data. In GC patients, the low survival rate has an insignificant relationship with levels of NEAT-1, according to a survival analysis. NEAT1 has been significantly downregulated in Isfahan samples of GC patients compared to the control, according to the analysis of 2 cm real-time PCR data. According to ROC analysis, for Isfahan patients with GC, NEAT-1 was found to be a good prognostic biomarker and a novel factor for distinguishing samples of control from samples of tumor (P-value 0.0001).

It has been shown that NEAT-1 levels and activity could be controlled by three non-coding and coding RNAs (MTRNR2L8, XIST, and hsa-miR-612). Microarray data analyses, ENCORI, and GEPIA2 were used for an expression study of coding and non-coding RNAs to comprehend their potential involvement in GC patients. MTRNR2L8 did not exhibit any appreciable low expression in the GC samples, according to the examination of the ENCORI data. In GC samples, there was no notable dysregulation according to GEPIA2 online expression analysis. Additionally, a GEPIA2 expression study showed that there is no link between the level of MTRNR2L8 and the stage of GC. In GC patients, the decreased survival rate and decreased levels of MTRNR2L8 were not significantly correlated, according to a survival study of the ENCORI and GEPIA2 datasets. According to the ENCORI analysis of relative expression of the lncRNA XIST, its upregulation in GC samples is not statistically significant (FC: 1.44, FDR: 0.74).

XIST has noticeably low levels in GC samples, according to GEPIA2 online analysis of expression. The results of a survival analysis showed no association between the expression pattern of the lncRNA XIST and survival rates of patients with GC and BC. As a result, the lncRNA NEAT1 can discriminate between tumor and control samples in the Isfahan population, making it a great diagnostic biomarker for individuals with GC (Azadeh et al., 2022). The expression of EZH2 was increased in GC by NEAT1, which increased the cell proliferation (He and Ren, 2024).

#### 3.5.2 N6-methyladenosine-related lncRNAs

One of RNA’s most prevalent internal modifications, N6-methyladenosine (m6A), regulates a number of bioprocesses, including the growth of cells, DNA damage response, and carcinogenesis (Rauch, 2020). Studies have demonstrated the significance of m6A alteration in the management of tumors, particularly in targeted therapy (Hu et al., 2019). The METTL3, METTL14, and WTAP m6A methyltransferase complex mediates the m6A modification, which is terminated by the FTO and ALKBH5, which are m6A demethylases. At the same time, reader proteins like YTHDC1, YTHDC2, and YTHDF1 are necessary for m6A modification (Fang et al., 2021). Three elements, namely: binding proteins (readers), methyltransferases (writers), and demethylases (erasers), make up regulators of m6A (Kong et al., 2022). Recent research has shown that either carcinogenesis is encouraged or the growth of a malignant tumor is inhibited by m6A. According to Yang et al.’s research, alteration of methylation mediated by m6A controls changes in the invasion of the microenvironment of the tumor (Yang et al., 2020). Modification of m6A improved immune regulation, which is mediated by YTHDF1. Malignant tumors may benefit from using YTHDF1 as a possible therapeutic target. Additionally, YTHDF1 is crucial for the immune evasion of tumors; however, more research is needed to determine the precise mechanism. Modification of m6A is crucial for cancer patient prognosis (Han et al., 2019).

There is a complicated involvement of changes in m6A in the biological function, development, and occurrence of cancer of the esophagus, and it represents a novel epigenetic research area. According to studies, patients with hepatocellular carcinoma and GC have much greater levels of m6A than healthy people do, and the amount is favorably linked with the clinical outcome (Lin et al., 2019; Wang Q. et al., 2020). Lower levels of m6A, however, are linked to a worse outcome in cases of bladder cancer (Gu et al., 2020). The discovery of the m6A modification on a number of non-coding RNAs, such as lncRNA, circRNA, mRNA, snoRNA, and microRNA, is the cause of this (Pan, 2013). It is important to note that the m6A alteration of RNA regulates the progression of oncogenesis and plays an essential function in innate immunity (Chen et al., 2019). RNA–protein interaction is impacted by the m6A alteration of lncRNA (Huang et al., 2020). Dot blot analysis evaluated the degree of m6A alteration in GC tissues. The findings demonstrated that 11 of 12 patient cancer tissues expressed more m6A than para-carcinoma tissues. Correlation analysis revealed that patient characteristics, clinical stage, and grade of tumor were higher with increased levels of m6A (P = 0.111, R = 0.485), particularly clinical stage (P = 0.048, R = 0.581). Because m6A writers frequently act as a tumor promoter during tumor growth, they are crucial for the development of cancer.

The matrix expression of 1,056 m6A-related lncRNAs and 23 m6A genes from the TCGA database was obtained. The m6A-related lncRNAs related to overall survival were screened and identified using a univariate Cox regression analysis. The candidate lncRNAs were divided into two categories: a protective type, which was related to good prognosis with HR < 1, and a risk type, which was related to a poor prognosis with HR > 1. Of these, eight lncRNAs (AC026333.4, SNHG3, SCAT2, MIR17HG, AC099850.4, AL512506.1, AL033527.3, and AC091057.1) were shown to be overexpressed in cancer tissues. The Consensus Cluster Plus package in R was used to divide the 72 patients who were a part of this investigation into two clusters. Two molecular subtypes were identified when k = 2. Patient prognosis was considerably different between the two subtypes. According to the overall survival findings, cluster 2 patients have a notably poorer prognosis than cluster 1 patients (P = 0.018). A notable difference exists between the clinical outcomes of the two subtypes. Potential biomarkers for predicting the overall survival of Asian patients with GC include N6-methyladenosine-related lncRNAs (Xu et al., 2022; Ji et al., 2025).

### 3.6 Circular RNAs (circRNAs)

CircRNA is a specific ncRNA type. The structure of CircRNA has covalently closed loops without poly-adenylated tails at the 3′ ends or a cap structure at the 5′ end. Compared to parental mRNAs, circRNAs have a significantly longer half-life (10 h vs. 48 h) and are capable of avoiding exonuclease destruction (Jeck and Sharpless, 2014). This conservative trait has led numerous studies to focus on the possible function as a favorable biomarker of a disease of circRNAs. They can interact with lncRNAs (long non-coding RNAs), RBPs (RNA-binding proteins), mRNAs, or sponging mRNAs to directly regulate transcription (Hansen et al., 2013; Memczak et al., 2013). Even the translation of some circRNAs into proteins is possible (Pamudurti et al., 2017). The circRNAs are transcriptionally back-spliced single-stranded ncRNAs (non-coding RNAs) that are generated from pre-mRNAs (Memczak et al., 2013). Considering their functional significance, they appear to primarily act as “miRNA sponges,” which allows the regulation of numerous growth regulatory genes that act as different miRNA downstream targets (Memczak et al., 2013; Panda, 2018; Li et al., 2020). It is intriguing to note that numerous circRNAs have recently been identified that are important in the pathogenesis of various human malignancies, along with GC (Li P. et al., 2017; Ma et al., 2020).

Due to the circular configuration of the circRNAs, they become resistant to RNase-mediated degradation and enable easy detection in tissue-specific and high-degree body fluids. They have attracted increasing attention from the perspective of clinical biomarkers (Xia et al., 2017; Wang Y. et al., 2020). CircRNAs are more stable in plasma and easier to detect than these conventional biomarkers. By sponging miRNAs, circRNAs can function as ceRNAs and create a circRNA-miRNA-mRNA network. Numerous studies have suggested that miRNAs, including miR-21, miR-7, and miR-145, play a role in EGFR mutation and consequently influence lung cancer invasion and metastasis (Chou et al., 2010; Chiou et al., 2015).

Recent studies have concentrated on the diagnostic utility of plasma circRNAs in combination with traditional markers, including PIVKA-II, AFP-L3, and AFP. According to Zhang et al., the plasma concentration of hsa_circ_0001445 could be used to efficaciously distinguish people who have cirrhosis, hepatitis, and liver cancer. Hsa_circ_0001445 of plasma had 94.2% specificity for HCC (hepatocellular carcinoma) diagnosis. Furthermore, a gradual logistic regression model revealed that the accuracy of a combination of AFP and has_circ_0001445 in HCC diagnosis was considerably better than either test alone (AUC = 0.970) (Zhang X. et al., 2018). Ye et al. confirmed that hsa_circ_0010882 expression was considerably increased in GC patients’ plasma, and it was favorably connected with differentiation of tumor and TNM stage. Additional research revealed that hsa_circ_0010882 in plasma interacted with the PI3K/Akt pathway to promote proliferation along with metastasis of GC cells (Peng et al., 2020). Li et al. designed a circular RNA panel using three circRNAs in plasma (hsa_circ_0005927, hsa_circ_000178, and hsa_circ_1900), which were upregulated (Peng et al., 2020).

The panel of circular RNAs showed significant advantages in differentiating colorectal cancer patients from control healthy individuals and demonstrated increased accuracy of diagnosis than CEA (AUC = 0.698) (Zhang et al., 2020; Zhou et al., 2021). Yin et al. used microarray technology and found 41 circRNAs that were expressed abnormally in BC patients’ plasma. The increased expression of plasma hsa_circ_0001785 demonstrated greater diagnostic resolution than CA15-3 or CEA (AUC = 0.784). Additionally, after surgery, the hsa_circ_0001785 plasma expression steadily decreased. Hsa_circ_0001785 in plasma may be useful for both diagnosis and treatment (Peng et al., 2020). Plasma circRNAs have also been investigated in different malignancies, along with a few cancers that were high in incidence. A poor prognosis and increased malignancy of gallbladder cancer were indicated by an increased level of circ-MTO1 in plasma (Wang et al., 2020c). In addition to enabling the early detection of GC patients, an 8-circRNA biomarker panel was developed, which demonstrated marked superiority in specificity and efficacy compared to the conventional tumor markers currently in use for the early diagnosis of gastric neoplasia.

First, to identify putative circRNA biomarker candidates, two genome-wide circRNA expression profile datasets from patients with GC were analyzed. Twenty-six downregulated and 53 upregulated circRNAs (adjusted P-value <0.05 and log2FC > 1) were found using differential expression analysis. Priority was given to circRNAs, which were overexpressed in GC tissues, as opposed to adjacent normal mucosa (ANM). In order to identify ten potential circRNAs, circRNAs with a log2FC > 2 and greater robustness of expression in the ANM vs. GC tissues were chosen. The results of the research showed that this panel of biomarkers had potential for robust diagnosis, with an AUC = 1.00 (P < 0.001 and 95% CI: 1.00–1.00), demonstrating its remarkable diagnostic ability to identify people who have gastric neoplasia.

The feasibility of converting the panel of biomarkers of circRNA, which is tissue-based, into a liquid biopsy was examined by measuring the expression of these biomarkers in blood specimens obtained from a training cohort of 46 healthy controls and 92 patients with GC. It was found that two circRNAs, hsa_circ_0055521 and hsa_circ_0004339, were not expressed in specimens of serum, and they were therefore eliminated from further study. Assays of RT-qPCR were used to determine the levels of the remaining eight circRNAs, and their diagnostic potential was analyzed. With an AUC of 0.87 (specificity 78.3%, sensitivity 78.3%, P < 0.001, and 95% CI: 0.82–0.93), the panel of 8-circRNA biomarkers was shown to distinguish individuals with GC from healthy controls. Univariate analysis was also carried out for the diagnosis of GC to examine the robustness of specific circRNAs. Six of the eight circRNAs showed a notable correlation in GC discrimination, while the P-values of the other two outcomes were less than 0.1. Given that surveillance by endoscopy is not a routine screening technique for this cancer in many nations, this is new and promising evidence regarding the importance of circRNA-based biomarkers for their use in clinical settings as high-throughput, reasonably priced, and non-invasive biomarkers for the identification of GC patients early using liquid biopsy (Roy et al., 2022).

### 3.7 Protein-coding gene-based biomarkers

Protein-coding genes serve as important biomarkers in GC due to their direct involvement in tumor biology. HAMP, a regulator of iron metabolism, is frequently upregulated in GC and associated with inflammation and tumor progression. PRTN3, a serine protease, is linked to immune modulation and has shown increased expression in GC tissues.

#### 3.7.1 HAMP

Hepatocytes in the liver produce hepcidin, a little polypeptide hormone that is essential for maintaining the proper balance of iron metabolism (Park et al., 2001). When high levels of circulating iron are needed and inadequate erythropoiesis takes place, diseases including anemia, hypoxia, and thalassemia result. This reduces the body’s ability to produce hepcidin (Ganz, 2003). Macrophages cause sequestration of iron, which leads to a reduction of the iron present in circulation. This defense mechanism results in inflammation and infection, during which hepcidin antimicrobial peptide (HAMP) expression increases. Conversely, during inflammation and infection, the expression of hepcidin antimicrobial peptide (HAMP) is increased because self-defense mechanisms result in a decrease in circulating iron due to the sequestration of iron by macrophages (Nemeth et al., 2004).

HAMP contributes to the development of different malignancies. Increased levels of circulating hepcidin have been linked to a variety of cancers, according to recent investigations. Serum hepcidin levels were discovered to be higher in people with breast cancer (Zhang S. et al., 2014). A higher tumor (T) stage was associated with greater HAMP expression seen in colorectal cancer tissues (Ward et al., 2008). High HAMP expression in lung cancer patients may affect prognosis via regulating immune infiltration (Fan et al., 2021). In comparison to low levels of HAMP expression, high levels were substantially related to a worse prognosis in assessments of overall survival (OS) in pancreatic cancer (Toshiyama et al., 2018). Hepcidin expression is extensive in the cells of prostate cancer and, by increasing intracellular iron transport, it can regulate cell division, migration, and death (Wang F. et al., 2017). In contrast, the expression of HAMP in cholangiocarcinoma and hepatocellular cancer was less than in adjacent normal tissues. A poor prognosis and an advanced pathological grade are linked to HAMP’s decreased expression (Shen et al., 2019; Wang and Du, 2021). Using experimental and bioinformatics tools, the degree to which HAMP was expressed in GC tissues was also validated. Furthermore, the results of one investigation showed a link between a GC patient’s prognosis, increased HAMP expression, and clinicopathological findings. Eventually, it was shown that there was a strong link connecting immunological infiltration and increased expression of HAMP.

To evaluate HAMP mRNA expression in GC, pan-cancer data that include 33 types of cancer in GTEx and TCGA were used. When compared with the normal tissues, the expression of HAMP was considerably higher in GC (P < 0.001). Using XIANTAO online tools, the results show that HAMP levels were higher in tumor samples (P < 0.001), which agrees with the earlier findings in 27 matched pairs of tumors and normal cells and tissue. Twenty-two of the GC patients had elevated HAMP expression. When compared to normal tissues, HAMP expression was considerably higher in GC (P < 0.001). The significance of HAMP expression was further assessed using 53 pairs of tissue samples and clinical data from gastric para-carcinoma and carcinoma. The findings revealed that Lauren classification and HAMP expression were related (P = 0.047), and other clinical features did not differ significantly. Due to the small sample size, the online tool XIANTAO was used to carry out additional validation in the TCGA database. According to a sex-based norm, the level of HAMP was higher in men and women. A higher TNM stage was correlated with increased HAMP levels and their upregulation. HAMP expression and upregulation were substantially correlated with the TNM stage when the TNM stage was higher. Additionally, compared to G0, HAMP levels were greater in histologic grades of G1, G2, and G3. HAMP overexpression is linked to poor FP, OS, and PPS in GC patients.

The relationship between the expression of HAMP and prognosis and tumor was then investigated. In patients with stage 3 GC, poor OS and FP were associated with increased HAMP expression, depending on the stage of the tumor. It was found that moderately differentiated tumors with elevated HAMP expression had poorer OS. In addition, both male and female GC patients with elevated HAMP levels had poor OS and FS. Following surgery only, increased expression of HAMP was additionally linked to poor OS and FS. The findings suggested a link between higher HAMP expression and adverse clinical outcomes. To investigate its importance and to suggest HAMP as a more favorable therapeutic target and diagnostic biomarker, publicly accessible data of gene expression were combined in GC patients with verification of the experiment. Additionally, it was discovered that immune infiltration in GC and HAMP expression were strongly correlated (Yang et al., 2022).

#### 3.7.2 PRTN3

PRTN3 encodes the neutrophil-derived protease proteinase-3 (PR3), which is the target of ANCA. Genetic variations at the locus of PRTN3 is linked to granulomatosis along with polyangiitis (GPA)/PR3-ANCA (Chen et al., 2023). PRTN3 expression (message) in healthy controls was shown to be associated with an eQTL (expression quantitative trait locus), rs62132293, which is a single-nucleotide polymorphism (SNP) found upstream of the PRTN3 transcription start site (Chen et al., 2023). Elevated PRTN3 gene expression (message) has an effect on the generation of circulating autoantigens, which may predispose individuals to serious illness. Although in healthy people, rs62132293 is a well-established eQTL for gene expression of PRTN3, no studies have been conducted to date on the effect of gene expression variants, clinical outcomes in patients, or circulating PR3 (Chen et al., 2023).

The *in vitro* labeling methods are known as isobaric tags for relative and absolute quantitation (iTRAQ) and were created by ABI, Inc. iTRAQ is frequently used in the analysis of differentially expressed investigations due to its benefits of high resolution, high throughput, a high degree of automation, accurate, and reproducible quantification using copious data (Li et al., 2021). iTRAQ was utilized to find GC biomarkers by employing a strategy of iTRAQ quantitative proteomics. It was found that from NGC vs. EGC, 502 DEPs were upregulated in gastric tissues, and from PGC vs. EGC, 727 DEPs were upregulated. RNA binding and innate immune responses are the main functions of DEPs. PRTN3 was found to differentiate early GC using gene ontology enrichment analysis. A PRM assay revealed that in the gastric mucosa of patients with EGC, PRTN3 was upregulated compared to patients with NGC and PGC. Hence, gastric mucosa PRTN3 acts as a novel diagnostic biomarker for assessing early GC patients.

A strict cutoff level of P-value <0.05 and log2 (fold change) = 1.2 was used to screen differentially expressed proteins (DEPs). When comparing NGC to EGC, PGC to NGC, and EGC to PGC, it was found that 281, 253, and 387 proteins were downregulated and 502, 213, and 727 proteins were upregulated, respectively. After that, EGC was compared to NGC and EGC to PGC using a Gene Ontology enrichment analysis. EGC differed from PGC and NGC through “ficolin-1-rich granule lumen” and “azurophil granule lumen,” according to the associations between the topmost six GO terms that corresponded to the enriched DEPs. Interestingly, in both analyses, PRTN3 was the most notable protein that was elevated. As a result, PRTN3 could be employed as a gastric mucosa marker for differentiating healthy, early-stage, and advanced-stage GC.

Each group’s differential expression was evaluated. In total, 3,928 proteins were found, from which 783 (502 upregulated and 281 downregulated) between NGC and EGC and 1,114 (727 upregulated and 387 downregulated) between PGC and EGC were DEPs. Compared to NGC vs. EGC and PGC vs. EGC, PRTN3 expression was noticeably increased, similar to the results from above. To further support the hypothesis, the protein expressions that were expressed differentially in each group’s gastric mucosa were quantified. In PGC vs. EGC and NGC vs. EGC, PRTN was the protein that was most elevated and had the highest FC value. In both the EGC vs. NGC and EGC vs. PGC comparisons, PRTN3 was the protein that was most elevated and had the highest FC value. There was no statistically significant difference in expression of PRTN3 between the groups of PGC and NGC compared to either group of PGC and EGC or the NGC and EGC groups. According to the findings, a significant increase in gastric mucosa PRTN3 levels may be a sign of early GC.

In conclusion, when comparing PGC and NGC patients, the gastric mucosal tissue PRTN3 expression was significantly higher in EGC patients. This suggests a novel endoscopic screening method to detect early GC (Guo et al., 2023). PRTN3 is upregulated in GC and correlates with advanced N stage. Silencing PRTN3 suppresses cell cycle progression, reduces GC cell proliferation (∼40%), and increases apoptosis (∼35%) (Zhu et al., 2025). Various clinical trials related to these biomarkers are included in Table 1.

**TABLE 1:** Clinical trials accompanied by various biomarkers in GC.

Biomarker	Type	Upregulation/downregulation	No. of patients	Location for sampling	Reference
HSPA6	Autosomal-recessive candidate protein	Upregulated	166	Tissue (gene)	Zhang et al. (2023b)
ANXA11	Calcium (Ca2+)-regulated phospholipid-binding proteins	Upregulated	255 subjects in two stages	Urine	Fan et al. (2022)
CDC42	Cell division control protein 42 of the Rho family of GTPases	Upregulated	255 subjects in two stages	Urine	Fan et al. (2022)
NAPA	Protein-coding genes	Upregulated	255 subjects in two stages	Urine	Fan et al. (2022)
FAP	A type 2 membrane-bound glycoprotein	Upregulated	56 normal samples and 60 GC samples	Differentially expressed gene	Li et al. (2022)
SERPINH1/HSP47	Serpin that serves as a human chaperone protein for collagen	Upregulated	56 normal samples and 60 GC samples	Differentially expressed gene	Li et al. (2022)
HAMP (hepcidin antimicrobial peptide)	Polypeptide hormone	Upregulated	53 patients	GC cells and tissues	Yang et al. (2022)
Cystatin B (CSTB)	A protein structure encoded by the CSTB gene	Downregulated	31 healthy individuals and 31 GC (adenocarcinoma) patients	Saliva	Koopaie et al. (2022)
Deleted in malignant brain tumor 1 (DMBT1)	A tumor-inhibiting gene	Upregulated	31 healthy individuals and 31 GC (adenocarcinoma) patients	Saliva	Koopaie et al. (2022)
Nuclear-enriched abundant transcript 1 (NEAT1)	lncRNA	Downregulated	21 control samples and 111 GC samples	Gene	Azadeh et al. (2022)
N6-methyladenosine-related lncRNAs	Internal modification in RNA	Upregulated	RNA sequence transcriptome data of 88 Asian GC patients 23 m6A-related genes were extracted from the TCGA database	Tissue (gene)	Xu et al. (2022)
8 circular RNAs (circRNAs)	Circulating non-coding RNAs	Upregulated	Two independent datasets (GSE89143 and GSE83521). All expression profiling data were downloaded from the Gene Expression Omnibus (GEO) database. A pilot cohort of 28 matched GC and adjacent normal mucosal (ANM) tissues. The training cohort consisted of 92 patients with GC and 46 endoscopically negative patients (non-disease controls) enrolled at Kumamoto University, Japan, between 2010 and 2015. The second validation cohort consisted of serum specimens from 102 GC patients and 48 non-disease control subjects enrolled at the Mie University Graduate School of Medicine, Japan, between 2006 and 2017. Independent cohort of 24 GC patients from March 2017 to March 2018 at Nagoya University, Japan.	Serum	Roy et al. (2022)
PRTN3	A serine protease enzyme expressed mainly in neutrophil granulocytes encoded by the PRTN3 gene	Upregulated	25 patients with early GC, 25 patients with progressive GC, and 25 control individuals with chronic gastritis	Tissue sample	Guo et al. (2023)

## 4 Multi-omics integration and combined biomarker panels in GC

The integration of multi-omics approaches combining genomic, epigenomic (e.g., DNA methylation), transcriptomic, proteomic, and metabolomic data offers a powerful framework for developing robust biomarker panels for early GC detection (Ali, 2023; Li et al., 2024a). Multi-omics panels consistently outperform individual markers by leveraging complementary molecular signatures, improving both sensitivity and specificity (Olivier et al., 2019). For instance, a recent liquid biopsy study (ASCEND-GASTRIC) combined cfDNA methylation, ctDNA mutation, and serum protein markers to show enhanced early detection capability for GC compared to single-layer analyses (Wang et al., 2023). A study used multiple dimensions of cfDNA, including fragment size, copy number variation, nucleosome coverage, and single-nucleotide patterns, to detect stage I–II GC, achieving AUROC values of 0.96–0.97 across validation cohorts (Yu et al., 2024). Broad profiling of cell-free multi-omics data in GC cohorts has also confirmed that integrated omics yield superior identification of cancer-specific signatures compared to single-omic models (Tao et al., 2023). Moreover, recent cohort-level studies augment these findings. The GutSeer assay combined targeted DNA methylation and fragmentomics sequencing in plasma to detect gastrointestinal cancers; while less sensitive for GC (∼65%), it achieved high specificity (95%–96%) in a large validation cohort, demonstrating the clinical feasibility of multi-omic panels (Huang et al., 2025).

Comprehensive multi-omics analyses incorporating mutations, CNVs, methylation, and gene expression from TCGA data enabled the creation of optimized prognostic models with increased accuracy (AUC ∼0.85) compared to single-omic counterparts (Guo et al., 2024). Lastly, multi-level omics reviews highlight the potential of integrating biological networks and machine learning to define early GC biomarkers from dynamic inflammatory cascades to malignant transformation (Zhang et al., 2024).

Together, these studies underscore the promise of multi-omics integration and combined biomarker panels for advancing early GC diagnosis. By harnessing complementary molecular signals and employing machine learning–based classification, this strategy increases diagnostic precision, sheds light on tumor heterogeneity, and may facilitate future translation into non-invasive clinical assays.

## 5 Conclusion and future perspectives

In recent years, the identification of novel biomarkers across multiple biological samples has significantly advanced the early diagnosis of GC, a disease often detected at advanced stages due to subtle initial symptoms. Biomarkers identified in saliva (e.g., CSTB and DMBT1), urine (e.g., ANXA11, CDC42, NAPA), tissue (e.g., HSPA6, m6A-related lncRNAs, and PRTN3), genetic profiles (e.g., FAP, SERPINH1, HAMP, NEAT1), and serum (e.g., various circRNAs) have demonstrated potential for improving early detection and guiding therapeutic strategies. These discoveries have laid the groundwork for more accessible, cost-effective, and non-invasive diagnostic solutions. Despite these promising developments, several critical challenges remain. Many of the reported biomarkers lack large-scale clinical validation, suffer from poor reproducibility across populations, or are limited by insufficient sensitivity and specificity when used alone. Additionally, the translation from bench to bedside is hindered by the lack of standardized protocols for sample collection, processing, and interpretation.

To bridge these gaps, future research should focus on integrating multi-omics data, employing artificial intelligence for biomarker panel optimization, and conducting prospective multicenter clinical trials. There is also a need to develop non-invasive, point-of-care diagnostic platforms, possibly using biosensors, radiolabeled agents, and advanced molecular imaging techniques. Such innovations could drastically reduce diagnostic delays and improve survival rates. Ultimately, a multidisciplinary approach combining molecular biology, bioengineering, computational science, and clinical research is essential to develop robust, reliable, and widely applicable biomarkers for the early detection and monitoring of GC.

## Abbreviations

HSPA6, Heat Shock Protein Family A6; ANXA11, Annexin A11; CDC42, Cell Division Cycle 42; FAP, Fibroblast Activation Protein-alpha, HAMP, Hepcidin Antimicrobial Peptide; SLC25A4, Solute Carrier Family 25 member 4; SERPINH1, Serpin Peptidase Inhibitor Clade H Member 1; DMBT1, Deleted in Malignant Brain Tumours 1; NEAT1, Nuclear Paraspeckle Assembly Transcript 1; PRTN3, Proteinase 3; NAS, Nitric acid synthase; ADC, Arginase decarboxylase enzyme; NO, Nitric oxide.
